# Evolution, expression and association of the chemosensory protein genes with the outbreak phase of the two main pest locusts

**DOI:** 10.1038/s41598-017-07068-0

**Published:** 2017-07-27

**Authors:** R. Martín-Blázquez, B. Chen, L. Kang, M. Bakkali

**Affiliations:** 1 Departamento de Genética, Facultad de Ciencias, Universidad de Granada, Fuentenueva S/N, 18071 Granada, Spain; 2 State Key Laboratory of Integrated Management of Pest Insects and Rodents, Institute of Zoology, Chinese Academy of Sciences, Beijing, 100101 China

## Abstract

We analyze the evolutionary relationships and expression patterns of the large set of genes for chemosensory proteins (CSPs) in the two main pest locusts. We used the available transcriptome and genome data to infer the number of genes using BLAST searches and sequence similarity matrices. Maximum likelihood phylogenies revealed the relationships between these CSPs and CSPs from several arthropods. RNAseq and qPCR allowed associating CSPs to locust phases. Crossing the phylogenetic and expression data allowed us to deduce homologies and conservation of the involvement in the phase change. We confirm that *Locusta migratoria* has at least 58 CSP gene copies, only five of which lack evidence of expression, and we reveal that *Schistocerca gregaria* has at least 42 expressed CSP genes. Both species share 21 orthologs, whereas 33 *L*. *migratoria* and 15 *S*. *gregaria* CSPs seem species-specific. Additional six *S*. *gregaria* and four *L*. *migratoria* CSPs seem duplications. Although the expression profiles are not especially conserved, seven orthologous CSP pairs share a gregarious over-expression pattern in adult locusts. We thus confirm that the number of locusts’ CSPs is large, due to gene duplications during the evolution of Orthoptera, we establish sequence and potential functional homologies, and we highlight specific CSPs that appear to be involved in locust gregariousness either in general or in a species-specific manner.

## Introduction

Locusts recurrently cause important economical losses and lead to famine in some of the most economically depressed areas of the globe. They show one of the most striking cases of phenotypic plasticity, referred to as phase polyphenism, with two mutually exclusive phenotypes: (i) a normal, low population density-induced, cryptic, sedentary and solitarious phenotype, and (ii) an occasional, high population density-induced, very active, conspicuous, swarming and gregarious phenotype^1–3^. The gregarious phase is triggered when thresholds of mechanical^4^, chemical^5, 6^ or visual stimuli^7^ are surpassed. As consequence, the locusts undergo drastic changes, including immediate physiological, metabolic and behavioural changes, colorimetric changes in the midterm and reproductive, developmental and morphological changes in the long term^1, 8–10^. For example, solitarious locusts develop gregarious behaviour if stimulated by friction of the hind femur^4^. Exposure to other individuals induces gregariousness even when the stimulus is olfactive only^9, 11, 12^. An example of the chemical triggers is phenylacetonitrile that is excreted by gregarious locusts as gregarizing pheromone^13, 14^ and male courtship inhibitor^15^.

The phase-dependent changes that the locusts undergo form part of a modulated response concomitant to the release and perception of pheromones and coupled to signalling via hormones and neuropeptides. Molecules such as dopamine^16, 17^, serotonin^12, 18, 19^ or corazonin^20–23^ have been reported as modulators of the locust phase change. Recent transcriptomics, genomics, methylation and metabolomics studies^24–30^ confirmed that the development of the gregarious phase involves several gene families and pathways (such as G protein coupled receptors, GPCR^31^), meaning that it is most likely polygenic and complex. Another interesting feature of the locust phase change is its dispersion along the orthopteran phylogeny^32, 33^ (a same genus may contain both polyphenic and non-polyphenic species).

Nonetheless, despite of its complexity, the phase change is density-dependent in all locusts. It is therefore reasonable to expect the molecules involved in perception of the environmental stimuli to be involved in triggering gregariousness in all locusts. These molecules constitute the first unavoidable contact zone between the outside and the insides of the organism, turning environmental stimuli into biological signals and cascades of interacting molecules and processes. Accordingly, several differences in stimuli receptors have been reported between solitarious and gregarious locusts. For example, olfactory sensilla are more abundant in solitarious *Schistocerca gregaria* individuals than in gregarious ones^34^, and injection of solitarious *S*. *gregaria* nymphs with corazonin led to gregarization and reduction of the number of antennal sensilla^20^.

Olfactory protein families such as the chemosensory proteins (CSPs) are among the molecules involved in the perception of environmental stimuli that have a high probability of being implicated in triggering locusts’ phase change. In fact, a CSP was found to bind 3-(1-naphthyl)propionitrile and 3-(2-naphthyl)propionitrile in *Locusta migratoria* ^35^, and over-expression of a CSP gene, *LmigCSP3*, has been reported in gregarious *L*. *migratoria*. Its knockdown led to a decreased ability to detect volatiles and to aggregate^36^. CSPs seem therefore to be part of the initial set of switches for turning the phase change on. They are a conserved family of soluble proteins, related to another family of chemoreceptors called odorant binding proteins (OBPs)^37^, and involved, as their name suggests, in chemoreception. Their number is variant between *taxa* and, while insect and mammalian OBPs are not related^38^, CSPs seem specific to Arthropoda^39, 40^. CSPs share a conserved amino acids pattern consisting of a predicted N-terminal signal peptide region, a conserved pattern of a cysteine followed by 6–8 residues, a second cysteine, 18 residues, a third cysteine, 2 residues then a fourth cysteine (Cys-*X*_6-8_-Cys-*X*_18_-Cys-*X*_2_-Cys)^41^. CSPs have six *α*-helices and their signal peptide might act as a transmembrane protein interactor that allows entry of the CSP into the endoplasmic reticulum for its later secretion. Disulfide bridges form between cysteines 1 and 2 and between cysteines 3 and 4 and the secondary structure of a CSP, in form of a globular protein with a ligand interacting cavity, is shaped by the position of its *α*-helices^42^. CSPs are eminently present in the haemolymph of the sensilla^43^, although they were also found to be expressed in other organs such as the ejaculatory bulb of *Drosophila melanogaster* ^44^, the legs of the cockroach *Periplaneta americana* ^45^, the embryonic states of the honey bee *Apis mellifera* ^46^ and the locusts’ neural and gonadal tissues^24, 36, 47–49^. Some CSPs therefore seem to have acquired novel biological functions, including in reproduction^44^, regeneration^45^ and development^46^.

Although their phylogenetic origin remains unsolved, CSPs very likely have followed a birth and death evolutionary dynamic^40^. Their number in insects is variable^40^, with some groups (like *Drosophila* genus) having a reduced number^50^, whereas others (such ants or butterflies) seem to have undergone an increase in the number of CSP copies in their genomes^51–53^. Five CSPs were reported for *L*. *migratoria* in ref. 48, and a GenBank search added 15 more CSPs for that species^47^. Later, a staggering number of 70 *L*. *migratoria*’s ESTs from the database were reported to be CSPs, and the expression of 17 of them was confirmed in the gonads^49^. In contrast, only five CSPs were hitherto reported for *S*. *gregaria* ^41^. Both sets of sequences were obtained by sequencing cloned cDNAs, meaning that they come from genuine expressed genes. Still, intra-specific comparison of the five *S*. *gregaria*’s and five *L*. *migratoria*’s CSP sequences casts high identity values^41, 48^, meaning that some of the inferred CSPs might be alleles of the same gene or gene copies with a very recent phylogenetic origin. The recent availability of locusts’ transcriptomics data^26, 27^ and a draft genome^29^ should allow further assessment of the number of *L*. *migratoria*’s CSPs and an approximation to that of *S*. *gregaria*. For the latter species, however, CSP detection can, for now, be based only on the available trancriptomics data from ref. 24 and from our own laboratory (Martín–Blázquez & Bakkali, in preparation), and a better estimation of their total copy number will have to wait until a genome assembly is available.

With CSPs being potential triggers, determination of which of them might be involved in locust gregariousness is of obvious relevance. It can be achieved in at least two ways. One is the functional genomics approach, based on knockdown of CSP genes in different individuals, stages and phases followed by in-depth examination of the knockdown phenotypes. Although it is direct, this approach would be laborious if not guided by preliminary data on what CSP to use and how, where, and when to observe the phenotype. An alternative approach could be based on phylogeny-guided comparison of the sequences and expression patterns of the CSPs of different locusts. While this latter approach is indirect and preliminary, it requires less preliminary knowledge on the function of the CSPs, is less laborious and its data might guide subsequent functional works.

Here we carry out a phylogenetic comparative analysis of the sequences and expression patterns of locusts’ CSPs. We compare the CSPs obtained from published works, databases and high throughput sequencing of the two most important locust species: *S*. *gregaria* (transcriptomic data) and *L*. *migratoria* (transcriptomic and genome sequencing data). Our first objective is to assess the number of CSPs in *L*. *migratoria* based on homology searches on its draft genome (version 2.4.1)^29^. We also identified all the transcribed CSPs from solitarious and gregarious transcriptomes of different *S*. *gregaria* tissues and compared them to those of *L*. *migratoria* in order to establish homologies between the CSPs of both species. A phylogenetic reconstruction using both locusts’ CSPs as well as several confirmed CSPs from other arthropods allowed us to identify different lineages and detect potential gene duplication events in the studied locusts. We also coupled phylogenies with differential gene expression between locust phases in order to check for parallelism between both characteristics of these locusts’ CSPs. The work thus allowed us to characterise the CSPs of the two most destructive locust species and to identify those whose differential expression patterns between phases is conserved in locusts. We hence infer on CSPs’ ancestry, specificity and importance for the development of the gregarious phase in locusts. The work also highlights particular CSPs for posterior functional testing.

## Results and Discussion

### The set of *L. migratoria’s* CSPs

**Figure 1:**
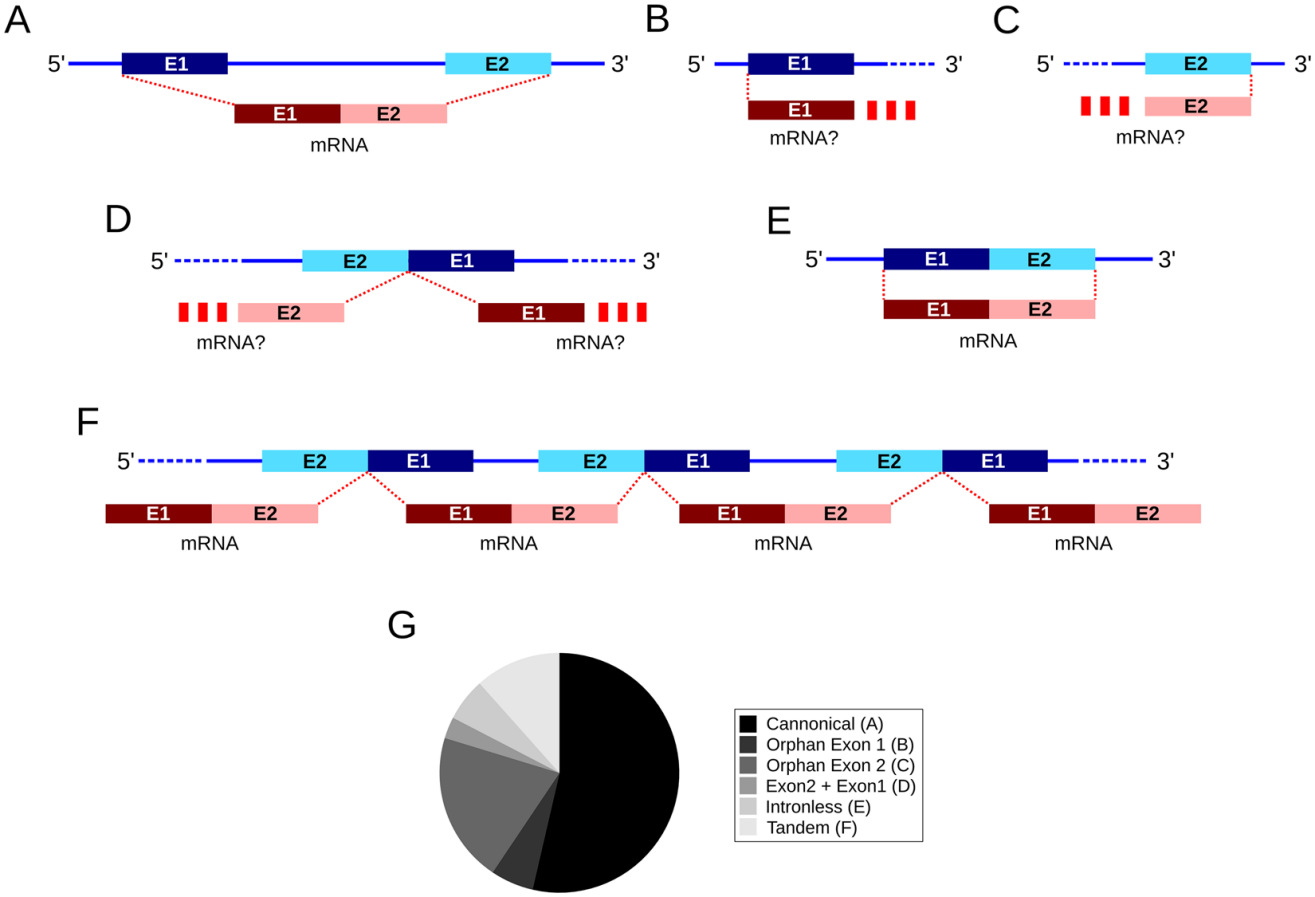
Schematic representation of each of the configurations shown by the CSPs’ exons in the different loci of *L*. *migratoria*’s draft genome version 2.4.1 (**A**–**F**). (**G**) Sector graph showing the relative prevalence of each of the configurations in (**A**–**F**).

Analysis of the list of CDSs in *L*. *migratoria*’s draft genome^29^ reveals 42 possible CSP loci located in 30 different scaffolds. For its part, tBLASTn search added seven loci in three additional scaffolds (Table 1). In addition to the canonical configuration of a CSP gene (Fig. 1A), we also detected four orphan exon 1 sequences (Fig. 1B), 14 orphan exon 2 (Fig. 1C), and two loci with exon 2 upstream of exon 1 (Fig. 1D). The mean length of the CSPs’ coding sequences (CDS) was 9050 ± 1459 bp, of which 8705 ± 1459 bp being intron. Four CSP loci contain no introns (Fig. 1E) and nine scaffolds contain more than one CSP gene, being eight the maximum number of CSPs found in a single locus—they are repeated in tandem in scaffold 71401 (Fig. 1F). Table S1 shows the genomic location and BLAST results for each CSP locus and Fig. 1G shows the prevalence of each genomic CSP structure.

**Table 1:** Location of the putative CSPs in the scaffolds of *L*. *migratoria*’s draft genome (version 2.4.1) and their best BLAST hit against the available ESTs from the same species.

Locus	Scaffold	Sense	Exon 1 start	Exon 2 end	Assigned ESTs
101	Scaffold 101	+	2366516	2400577	—
103059	Scaffold 103059	+	2171	10788	—
12585	Scaffold 12585	−	179306	167247	ORF16
13671	Scaffold 13671	−	595509	590973	—
**15810**	Scaffold 15810	−	78980	63220	**LmigCSPII-6**, LmigCSPII-8, LmigCSP2, LmigCSPII-7
18858cds1	Scaffold 18858	+	97840	108678	ORF17
18858cds2	Scaffold 18858	+	141703	149698	*LM*_*GH5*_*000761*
18858cds3	Scaffold 18858	+	168506	170451	—
**21551**	Scaffold 21551	−	122154	97824	**LM_SH5_001382**, ORF9
22826cds1	Scaffold 22826	−	159147	153386	*LM*_*SH5*_*003413*
**22826cds2**	Scaffold 22826	+	127283	129558	**LmigCSPII-11**, LmigCSPII-9, LM_GH5_003489, LM_SL5_002526, LM_SL5_002527
**235750**	Scaffold 235750	+	7652	9847	**LmigCSPII-14**, LmigCSPII-12
**24400**	Scaffold 24400	+	13865	18627	**LM_GH5_000758**, LM_GH5_000759, LmigCSPI-2, LmigCSPI-6, LmigCSPI-3
25611	Scaffold 25611	+	14519	63192	—
2564	Scaffold 2564	+	89690	97425	—
30358	Scaffold 30358	−	35537	22421	—
31810	Scaffold 31810	−	78016	67182	—
**320887**	Scaffold 320887	+	589	33184	**LM_GH5_003053**, ORF7
3212cds1	Scaffold 3212	−	1325340	1315951	—
3212cds2	Scaffold 3212	−	1382008	1363494	—
325580	Scaffold 325580	+	830	24951	LM_GB5_001536
33302cds1	Scaffold 33302	+	4672	5022	—
33302cds2	Scaffold 33302	+	10024	10374	—
37289	Scaffold 37289	+	4533	10346	ORF1
374630	Scaffold 374630	+	5695	6078	—
392768	Scaffold 392768	+	61	21224	ORF10
41553	Scaffold 41553	+	66870	72899	ORF14
46375	Scaffold 46375	+	44829	62108	—
5214cds1	Scaffold 5214	−	122926	116531	—
5214cds2	Scaffold 5214	+	143789	145156	—
57579	Scaffold 57579	+	3592	15413	—
**647**	Scaffold 647	−	198201	176012	**LM_GL5_000034**, LM_GL5_000035, ORF11, LM_GL5_000033
699cds1	Scaffold 699	+	80447	89313	ORF8
699cds2	Scaffold 699	−	152813	144007	—
71401cds1	Scaffold 71401	−	45335	44165	*LM*_*GH5*_*002985*
71401cds2	Scaffold 71401	−	44176	41897	LM_GH5_002985
71401cds3	Scaffold 71401	−	41890	40695	*LM*_*GH5*_*002985*
71401cds4	Scaffold 71401	−	40637	38659	*LM*_*GH5*_*002985*
**71401cds5**	Scaffold 71401	−	38626	35392	**LM_GH5_002985**, ORF18
71401cds6	Scaffold 71401	−	35385	33956	*LM*_*GH5*_*002985*
71401cds7	Scaffold 71401	−	33949	31212	LM_GH5_002985
71401cds8	Scaffold 71401	−	30988	28604	*LM*_*GH5*_*002985*
757cds1	Scaffold 757	−	3626	2064	LM_SH5_003270
**757cds2**	Scaffold 757	−	60814	58709	**LmigCSPII-10**, LmigCSP4, LmigCSP5, LM_GH5_003478
**757cds3**	Scaffold 757	−	95948	91849	**LM_SH5_003782**, LM_GH5_003820, LM_SH5_003512, ORF3, LmigCSP3, LM_GH5_003822, LM_GB5_007735
75957	Scaffold 75957	−	4048	3689	—
78016	Scaffold 78016	−	21591	16775	—
9174cds1	Scaffold 9174	+	10000	11523	LM_SH5_003244
9174cds2	Scaffold 9174	+	36759	38397	LmigCSPII-13
178632750	C178632750**	+	20	175	LM_GH5_003055
**187757636**	C187757636**	+	479	655	**LM_GB5_004555**, ORF19
50720***	Scaffold50720*	−	1643	1521	**LM_GH5_003725**, ORF5, LM_SH5_003413, LM_SH5_003651, ORF4
401450***	Scaffold401450**	+	218	412	
**53850**	Scaffold53850**	−	4232	4044	**LM_GH5_003400**, ORF2
68729	Scaffold68729**	−	7415	7227	LM_GH5_000760
**281155**	Scaffold281155**	+	5573	5761	**LM_GH5_000761**, ORF12
157799226	C157799226*	−	98	6	LM_SH5_003268
—	—	—	—	—	LM_GM5_003208
—	—	—	—	—	ORF13

This table shows all the retrieved sequences (complete genes, orphan exons and ESTs whose loci are not sequenced yet). They all have positive BLASTx results against CSP proteins from the NCBI database. A sequence was considered to be a putative locust CSP if it had the conserved pattern of four cysteines (see the main text), was composed of two exons (1 and 2) belonging to the same scaffold and in the correct relative position and orientation. *The locus has more than one allele (see Tables S1 and S2 for additional BLAST data and further data on the attribution of ESTs to genomic loci and to known CSPs). In bold are the multi-allelic genes and the ESTs that had the best BLAST against them. In italic are the ESTs whose significant blast result did not pass the established sequence identity threshold (see Methods). *Orphan exon 1, **Orphan exon 2, ***Orphan exons 1 and 2 that belong to different scaffolds but have significant hit against the same EST. The orphan exons that we couldn’t assign to any EST are in Table S1, and the ESTs that we couldn’t assign to any locus are in Table S2. The three consecutive dashes signal no available data.

The difference between the number of CSPs that we report for *L*. *migratoria* thus far, at least 55, and the 70 reported by Zhou *et al*.^49^ could be due to the incomplete state of the genome sequence and the fact that, thus far, we didn’t consider ESTs. Indeed 96 *L*. *migratoria*’s ESTs from Kang *et al*.^27^ have significant BLAST results against arthropods’ CSP proteins. The sequences of 34 of these contain the conserved CSP cysteine pattern, were not redundant (not identical), and were different from the 5 ESTs reported by Picimbon *et al*.^48^, the 15 ESTs from Ban *et al*.^54^, and from 19 non-redundant ESTs out of the 70 reported by Zhou *et al*.^49^. We discarded a sequence from the latter work (ORF15, accession number AJ973429.1) because we found it to have a disrupted (five) cysteine pattern. We therefore thus far detected 72 (34 + 5 + 15 + 18) CSP ESTs for *L*. *migratoria*. However, potential overlaps between the sets of genomic loci and ESTs may still exist. BLASTn of the ESTs against the genomic sequences (complete genes and orphan exons) solved the question and showed that 20 complete genes and 7 orphan exon 2 had no significant BLAST hit to any EST (Table S1). While, for precaution, we discarded the seven orphan exons for being incomplete and lacking further evidence in favour of a genuine CSP nature, the complete sequences were considered as CSP genes for which we do not have evidence of expression. The remaining 29 complete genomic sequences, 4 exon 1 and 14 exon 2 have best significant hits against 28 different ESTs (Tables 1 and S1). The difference between the numbers of ESTs and loci is due to the different (duplicated) genomic sequences that correspond to the same ESTs.

However, the set of 72 ESTs could also contain alleles of the same genes and ESTs whose genomic locus has not been sequenced yet. Effectively, reciprocal BLASTn of the ESTs against the genomic sequences revealed that 53 ESTs have best positive BLAST result against 20 different (not duplicated) genes—all these ESTs seem thus alleles of 20 expressed genes. 16 ESTs have their best positive BLAST results against orphan exons (three against two orphan exon 1, 12 against seven orphan exon 2, and one against an orphan exon 1 and an orphan exon 2)—hence these 16 ESTs appear to be alleles of 10 (2 + 7 + 1) expressed genes (Table S2). The remaining three ESTs show no significant BLAST result against any genomic locus (Table S2). The high (99.7%) nucleotide sequence similarity between two of them (ORF6 and LM_GM5_003208) suggests that they are alleles of the same unsequenced gene, while the third (ORF 13) seems to belong to a different gene (58.2% and 59% identity between ORF13 and ORF6 and LM_GM5_003208, respectively). Two are therefore the ESTs that correspond to unsequenced genes. Thus, the total number of *L*. *migratoria*’s putative CSPs appears to be 58 distributed as follows: 29 completely sequenced and expressed genes, 20 completely sequenced genes with no evidence of expression, four expressed genes whose exon 1 is still missing from the draft genome, two expressed genes whose exon 2 is still missing from the draft genome, a still not assembled expressed gene whose exon 1 and exon 2 belong to different scaffolds of the draft genome, and two expressed genes whose genomic loci are still not sequenced. We see therefore how combining transcriptome and genome data is not only useful for a more complete and accurate detection of the set of genes of a protein family, but also allows assessing the state of completion of a genome sequencing project (proportion of partially sequenced or unsequenced loci whose expression is confirmed) and might help genome assemblies by relating scaffolds based on their exon content (here we see how scaffold 50720 of *L*. *migratoria*’s draft genome version 2.4.1 must be located immediately downstream of scaffold 401450—since *LmigCSP1* exons 1 and 2 are in scaffolds 50720 and 401450, respectively).

### The set of *S. gregaria’s* CSPs

Only 11 contigs out of the few available *S*. *gregaria* genomic sequences from ref. 55 potentially contain CSPs. However, we discarded those contigs due to unreliable sequence patterns (Table S3). We thus relayed only on transcript data for that species. 179 non-redundant (not identical) *S*. *gregaria*’s transcripts have significant BLASTx against arthropods’ CSP proteins and contain the conserved CSP cysteine pattern. Five of these were already reported in ref. 41, nine were from the ESTs in ref. 24, and the remaining 165 were from our RNA-seq data (Table S4).

Such a large number of potential CSPs could be due to the presence of false positives, since some transcripts might be alleles of the same genes. We detected these based on phylogenies and sequence similarity thresholds inferred from *L*. *migratoria*’s CSP alleles. It is, to our understanding, the most objective way to distinguish between alleles (sequences that differ no more than an estimated similarity threshold) and genes (sequences that differ above that threshold). The phylogeny of *L*. *migratoria*’s CSP genes and transcripts (Fig. S1) shows that in addition to the genes that have no associated transcripts or are too closely grouped (i.e. recently diverged) as to unequivocally attribute them to transcripts, five clades contain a single genes and a single transcript and seven clades contain a single gene and various transcripts (alleles). The lowest pairwise identity between the sequences of each of these clades, 93.7% (Table S5), allowed us inferring which of the 179 *S*. *gregaria*’s ESTs correspond to different, “good”, CSP genes and which are alleles of the same genes. *S*. *gregaria* appeared to have 42 different CSP transcripts (marked in bold in Table S4).

Another potential issue with the *S*. *gregaria*’s CSPs reported here, which were produced by *de novo* assemblies, is whether they include chimera. We solved this issue based on congruency of the sequence similarities between the exon 1 parts and between the exon 2 parts of the CSPs. A *S*. *gregaria* CSP whose exon 1 and exon 2 parts appear similar to respective exons from different *L*. *migratoria* CSPs within or above an estimated threshold would be considered as chimeric result of a misassembly. We therefore analyzed the two exons separately. While exon 1 is more variable in length (80 to 242 bp; mean length = 167.755 ± 5.047 bp), exon 2 is larger (113 to 290 bp; mean length = 179.694 ± 4.247 bp). The maximum likelihood phylogenies of the nucleotide sequences of each exon (including those of *S*. *gregaria*’s transcripts and of *L*. *migratoria*’s genes and orphan exons) produced congruent clades except for the exons of six *S*. *gregaria*’s transcripts (about 15% of the total) that occupied incongruent positions in the two single-exon trees (Fig. S2A,B). The highest sequence identity between exons 1 and between exons 2 of the distinct *L*. *migratoria*’s CSP genes (defined as CSPs at different loci, see Methods) were 97% and 95%, respectively. No exons 1 and 2 of the same incongruent transcript simultaneously showed higher identities to their nearest neighbour sequences in the respective exon tree than these values. Hence, none of the above-mentioned six *S*. *gregaria*’s transcripts could be considered as chimeric (details in Table S6).

### Are the gene sets identified here to be trusted

The first issue relating to the question stated above is whether the CSPs that we report here are genuine nor not. The answer seems positive since we based our search and detection of these sequences on strict, clear and logical rules that used objective cut-offs. They included: (*i*) significant BLAST hit to CSP proteins, (*ii*) presence of the conserved CSP cysteine pattern, (*iii*) presence of the two CSP exons, (*iv*) concordant relative position of the two exons, (*v*) concordant relative orientation of the exons, (*vi*) concordant best BLAST result of each of the two exons against the same CSP protein, (*vii*) congruent phylogenetic position of each of the two exons of the same CSP and (*viii*) adequate sequence similarity thresholds. In fact, it is relevant that the number of non-redundant *L*. *migratoria*’s CSP genes (when we exclude redundancy from the duplicated genes) is similar to the number of CSP ESTs that we identify in *S*. *gregaria* (41 *vs*. 42, respectively). Furthermore, our methodology shows how CSPs that were hitherto considered as different genes (*e*.*g*., *LmigCSP5* and *LmigCSPII*-*10*) seem to be alleles of the same gene.

The other issue is the completeness of the set of CSPs that we report here. Beyond the numbers of detected sequences, the validity of our interpretation depends on the nature and amount of raw data as well as on the logic and search methods that we used. *In silico* detection of a full set of genes depends on the state of completion of the genome and on the presence of features that allow identifying the genes in question. CSPs have distinctive features that include the presence of two exons and, more importantly, a conserved pattern of four cysteines^41, 42, 44, 47, 56, 57^. However, only a tiny fraction of *S*. *gregaria*’s genome is presently covered^55^ and *L*. *migratoria*’s genome is only nearly completed^27^. To mitigate this, we complemented the available genomic and EST data using RNAseq data. Still, the use of transcriptome data only might present several limitations. Detection of the full set of CSPs from a single transcriptomics project is impossible and the number of detectable sequences depends on the material, timing and conditions of the experiment, as well as on the levels of gene expression, RNA handling, and on the sequencing method and depth. Furthermore, transcript-based searches, both *in vitro* (cloning and sequencing) and *in silico* (transcriptomics), might produce false positives (alleles of the same gene). Our solution was to search as much data as possible in order to detect the most complete set of genes. We carried out an exhaustive analysis of all the EST, genome, protein and NGS sequences of the public databases, as well as our over 500 million Illumina Hiseq. 2000 Paired End sequencing reads. The number of potentially undetected CSPs should thus be very likely low (a single digit), and the set of locusts’ CSP genes that we report here seems therefore almost complete and very likely lacks redundancies and false positives.

### Evolution of locusts’ CSP genes

Here we aim at understanding the evolution of a large set of sequences that belong to large genomes and are subjected to functional and selective constrains imposed by episodic exposure to extremely high population densities.

With at least 58 CSPs in *L*. *migratoria* and confirmed ESTs from 42 CSP genes in *S*. *gregaria*, locusts have more CSPs than other insects (*e*.*g*., *A*. *mellifera*, *A*. *gambiae* and several Drosophila species, see ref. 40), including those with confirmed duplication and diversification of CSPs, such as *B*. *mori* (21 CSPs^51^) and *T*. *castaneum* (19 CSPs^58^). Even ants have only 11 to 21 CSPs depending on the species^52^. In fact, in spite of Orthoptera having some of the biggest metazoans genomes^59^, the two locust species studied here are not polyploid. Expansion of CSP genes in their genomes must therefore have happened by gene duplications. The DNA of the CSP genes does not show relevant repetitions that might easily explain such duplications. Still, we found tandem repetitions, meaning that unequal crossing-over and recombination between homologous chromosomes, followed by selection or drift, could have contributed to part of the expansion of the CSP genes in locusts’ genomes. There are no clear footprints of CSP transposition, and CSPs are no transposable elements. Nonetheless, the presence of highly similar CSPs in different parts of the genome suggests that some movement of the CSPs between different loci may have happened—with such large genome sizes, genome-wide reorganizations may have taken place more frequently in locusts than in other species^60^. Comparison of the nucleotide sequence similarity (Tables S7 and S8) suggests that duplication of the three tandemly repeated paralogs in scaffold 757 is older than that of the three paralogs in scaffold 18858 which, in turn, seems older than that of the eight paralogs in scaffold 71401—some of which seem indeed very recent (paralogs 4, 5, 6 and 7).

BLASTn searches showed that *S*. *gregaria*’s CSP transcripts have at least one significant BLAST hit against 25 *L*. *migratoria*’s CSP genes (Table S4). Accordingly, a nucleotide phylogeny (Fig. S2C) shows 25 *L*. *migratoria*’s CSPs grouping with 27 *S*. *gregaria*’s CSPs in 21 inter-specific clades. 11 of these clades contain an orthologous pair of sequences, 6 contain a *L*. *migratoria*’s ortholog and two *S*. *gregaria*’s paralogs, and 4 contain a *S*. *gregaria*’s ortholog and two *L*. *migratoria*’s paralogs. The remaining 33 *L*. *migratoria*’s and 15 *S*. *gregaria*’s CSPs form species-specific clades. Species-specific expansions of the CSPs must therefore have occurred.

In addition to the conserved cysteine pattern, alignment of *L*. *migratoria*’s (Fig. S3) and *S*. *gregaria*’s (Fig. S4) CSP amino acid sequences reveals several conserved regions and a higher variability of the N-terminal region. ProtTest suggested LG + I + G^61^ as optimal amino acid substitution model, and the topology of the resulting maximum likelihood tree (Fig. S2D) was almost identical to that of the nucleotides tree, with no sequences occupying incongruent positions between trees. The amino acid tree shows overall shorter branch lengths, compared to the nucleotide tree, probably due to the presence of more synonymous than non-synonymous mutations.

The nucleotide sequence diversity (Table S9) between CSP orthologs, CSPs of the same species and even between CSP paralogs (same species and same phylogenetic clade) is, for instance, much higher than that reported for the *cis*-regulatory sequences of *Drosophila*’s *fushi tarazu* gene^62^ (0.045–0.505 *versus* 0.001–0.008, respectively). This might be due to divergence of some CSPs or to functional relaxation, or loss of function, of some redundant (phylogenetically related and duplicated) CSPs. Loss of function seems in agreement with the fact that only one transcript (LM_GH5_002985) associates with the eight juxtaposed CSP copies in scaffold 71401. However this is no tangible argument as possible transcripts of seven of these duplicated paralogs might have been filtered out as alleles of the same gene during the sequence editing processes employed in ref. 27. In fact, the LM_GH5_002985 EST contains an exon 1, an exon 2, and another exon 1—proof of its transcription from two CSP genes. This also raises questions about the post-transcriptional editing of the pre-mRNAs from tandemly repeated genes and its potential effect on the diversity of the resulting proteins.

Despite the high nucleotide diversity, K_a_/K_s_ values indicate more synonymous than non-synonymous substitutions per site (Table S8)—which explains the branch length differences between the congruent amino acid and nucleotide trees. Locusts’ CSPs hence seem to be under purifying selection, although the high standard deviations imply considerable differences between homologous pairs. The K_a_/K_s_ value is marginally lower for *S*. *gregaria*’s CSPs than for *L*. *migratoria*’s, probably because the former are transcripts (all functional) whereas the latter contain genomic sequences (not necessarily all of them functional). More importantly, the K_a_/K_s_ values of the paralogs at the same genomic locus are not higher neither than those of the paralogous or orthologous CSPs that share the same phylogenetic clade but not the same locus, nor than those of the sequences that share neither phylogenetic clade nor locus (Tables S8 and S9). Signs of purifying selection are therefore evident for the duplicated CSPs in the multi-CSP loci—suggesting conservation of function. The K_a_/K_s_ values of the orthologs are lower than one, suggesting conservation of amino acid sequences, hence of the function, and the particularly low value between *SgreCSP19* and *LmigCSP19* suggests a conserved and important function of that CSP for locusts (Table S9). As expected, the CSPs that share neither locus nor phylogenetic clade show K_a_/K_s_ values above one due to their sequence and functional divergence.

**Figure 2:**
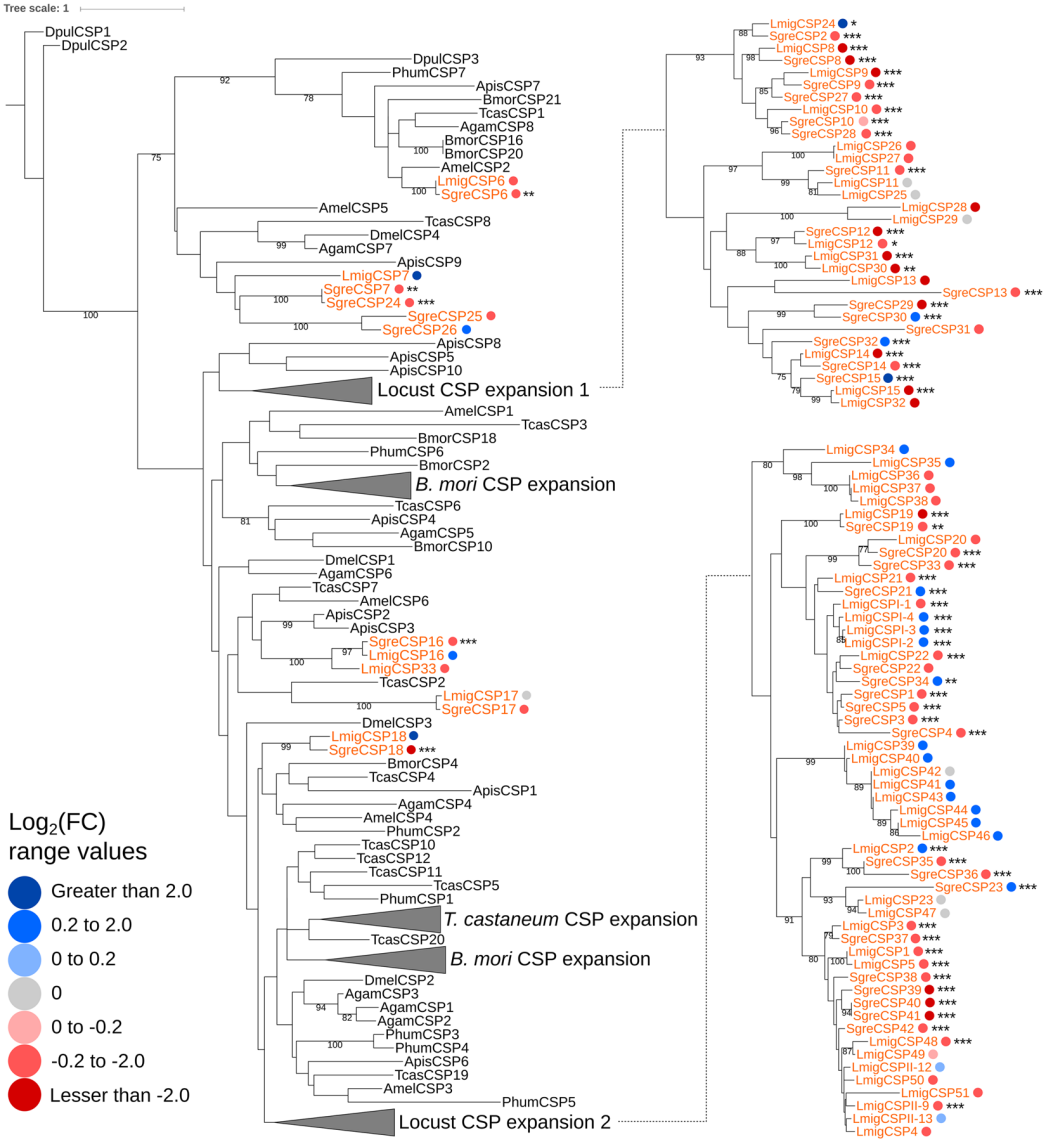
Maximum likelihood phylogenetic tree of the amino acids sequences of CSPs from multiple arthropod species. Locusts’ CSPs are in orange, and the large clades are compacted in grey triangles and developed in the right-hand side of the figure when necessary. The branch lengths follow the scale shown in the figure. Branch supports are shown if higher or equal to 75%. The solitarious *versus* gregarious expression levels, calculated as 2-based logarithm of the fold change (FC) between gregarious and solitarious libraries from adult bodies with no digestive tube (see Methods), are indicated using colored dots. Blue dots indicate over-expression in the solitarious phase and red dots indicate over-expression in the gregarious phase as shown by the color scale in the figure. The significance level of the over-expression, after FDR correction, is indicated using asterisks (*0.05–0.01, **0.01–0.001 and ***0.001–0). Note that locust CSP expansion 1 is more ancestral than expansion 2.

CSPs evolve following a birth-and-death dynamic^40, 50, 52^, and the number of their ancestral groups in arthropods was estimated to be seven, two of which showing high mutation rates^52^. A maximum likelihood tree of CSPs’ amino acid sequences from multiple arthropod species agrees with that interpretation as it shows seven clades, two of which being major locust-specific expansions that include 87 of the 100 locusts’ CSPs (Fig. 2). The ancestral expansion includes 17 CSPs from *L*. *migratoria* and 15 from *S*. *gregaria*, of which nine pairs are orthologs. The other, more recent, expansion contains 35 CSPs from *L*. *migratoria* and 19 from *S*. *gregaria*, of which seven pairs are orthologs. Of the six *L*. *migratoria*’s and eight *S*. *gregaria*’s CSPs that do not belong to any of these expansions, five pairs are orthologs. Thus, *L*. *migratoria* and *S*. *gregaria* share a total of 21 orthologous pairs of CSPs. Overall, arthropods’ CSPs are not especially conserved (Fig. S3I); as only 1.3% of their amino acid alignments’ sites are completely conserved, and their pairwise amino acid identities range between 24% and 40%. Accordingly, the 3.5% and 2.3% site conservation and 40.3% and 33.9% pairwise identities in *L*. *migratoria*’s and *S*. *gregaria*’s amino acid alignments, respectively, suggest that locusts’ CSPs are no exception in that respect (Figs S4 and S5). Still, in addition to the conserved cysteine region, CSPs of the terrestrial arthropods show three conserved regions (Fig. S5).

Strikingly, the sequence similarities and phylogenies show no clear orthology between CSPs that were reported in different works under similar names. We thus alert of the misleading incongruences in CSP numbering. For instance, *LmigCSP3* seems orthologuos to *SgreCSP37* while *SgreCSP3* seems orthologous to *LmigCSP22*. Similarily *SgreCSP2* seems orthologous to *LmigCSP24* while *LmigCSP2* seems orthologous to *SgreCSP35*. That’s why we used and suggest a phylogeny-guided naming rule based on a four-letters genus and species code (*Sgre* for *S*. *gregaria*, *Lmig* for *L*. *migratoria*, etc.) followed by CSP and a number. The number would be that of the ortholog, if any, or the next available number to that of the phylogenetically adjacent CSP (Table S10). We suggest that rule for naming the CSPs that future works might detect and, to avoid further confusion, we recommend adopting the chronologically first name given to an already named sequence instead of adding new names.

### Expression of the CSPs and its association with locust phases

Given CSPs’ function as receptors of environmental *stimuli*, we expect them to be associated with locusts’ phase change either by triggering it, maintaining it, and/or being affected by it. For that, the noticeable expansion of CSP genes in locusts’ genomes may be relevant—as is the case, for instance, with the expansion of the stress-related genes in tardigrades^63^. It is true that locusts have less CSPs per genome size than some other insects (see Table S11). However, what matters in functional terms is the number of genes and their expression, not their density in the genome. In fact, not all locusts’ genes have multiple copies, so only those whose DNA or function allow or require expansion have experienced expansion.

All the CSPs that we report here for *S*. *gregaria* are likely functional, as they are ESTs and assembled transcripts from RNAseq libraries of the central nervous system, digestive tube, muscle and testicles—the low expression levels of the CSPs in the ovaries (see below) did not allow the assembly of any valid CSP transcript from the reads of that tissue’s sequencing library. For *L*. *migratoria*, however, we report both ESTs and genomic sequences, 20 of the latter with no detected transcript. Since the names of *L*. *migratoria*’s ESTs from ref. 27 contain a code referring to the tissue from which they came, we could infer that 22 CSPs were expressed in the head, five in the hind legs and two in the midgut of *L*. *migratoria*’s 5^th^ instar nymphs. The remaining five were expressed in 5^th^ instar female nymphs (see Table S2).

22 of the 58 *L*. *migratoria*’s CSPs (38%) and 38 of the 42 *S*. *gregaria*’s CSPs (90%) show significant differential expression between phases in adults (Fig. 2). However, we cannot conclude that more CSPs are associated with the phase change in *S*. *gregaria* than in *L*. *migratoria*, since our sequencing libraries included *S*. *gregaria*’s CNS-enriched tissues whereas the available data on *L*. *migratoria* do not include such tissue enrichment. In fact, the CNS shows the highest expression levels of CSPs (Fig. S6B) while ovaries show the lowest levels (29 mapped reads maximum)—details in Fig. 4 and Table S12. Given the sensorial functions of the CSPs and the essential involvement of the CNS in locusts’ phase change, the higher expression and more pronounced differential expression of the CSPs in the CNS, compared to other tissues (Fig. S6B), is reasonable and explains the between-species differences in the numbers of CSPs that we found differentially expressed between phases.

Overall, CSPs show higher expression levels in the gregarious phase both in the analyzed *S*. *gregaria*’s tissues and throughout *L*. *migratoria*’s developmental stages (Fig. S6). Still, our CNS library is enriched with transcripts from antennae, palps and other sensory organs. It is therefore the most adequate material for studying CSPs, and locusts’ phase change in general. The fact that 40 out of the 42 *S*. *gregaria*’s CSPs show differential expression between phases in the CNS means that most of that locust’s CSPs are involved and/or affected by the phase change at least in the CNS. Even more, the fact that 36 out of these 40 differentially expressed CSPs are over-expressed in the gregarious phase is concordant with the increased sensorial inputs during that phase than during the solitarious one. Such tendency seems general to locusts as, overall, 37% of the differentially expressed CSPs in *S*. *gregaria* and a congruent 40% in *L*. *migratoria* show higher expression in the gregarious phase. No CSP shows conserved over-expression in solitarious *S*. *gregaria* and *L*. *migratoria*—a *datum* in accordance with the expected little need for detection of *stimuli* in low-density populations.

Like the ovaries, the testicles show almost no CSP expression (46 mapped reads maximum) except for one, *SgreCSP14*, with 5249 mapped reads and no differential expression between phases. The testicle tissues whose RNAs we sequenced include the ejaculatory bulb, and it is known that Ejaculatory Bulb Protein 3 (EBP3) is a CSP homolog^44^. However, *SgreCSP14* is not an EBP3 ortholog, as neither *SgreCSP14* nor its homolog *LmigCSP14* appear in EBP3-containing clades of the amino acid tree (Fig. S7). However, the position of the EBPs in the phylogeny is so different between species and strikingly close to specific CSPs that their distinction from CSPs and inference on the EBP as opposed to CSP nature of a sequence cannot be made based on sequence similarity. Whatever the case, what we know is that *LmigCSP14* binds phenylacetonitrile in *L*. *migratoria*’s testicles^35^; *SgreCSP14* should therefore do the same.

**Figure 3:**
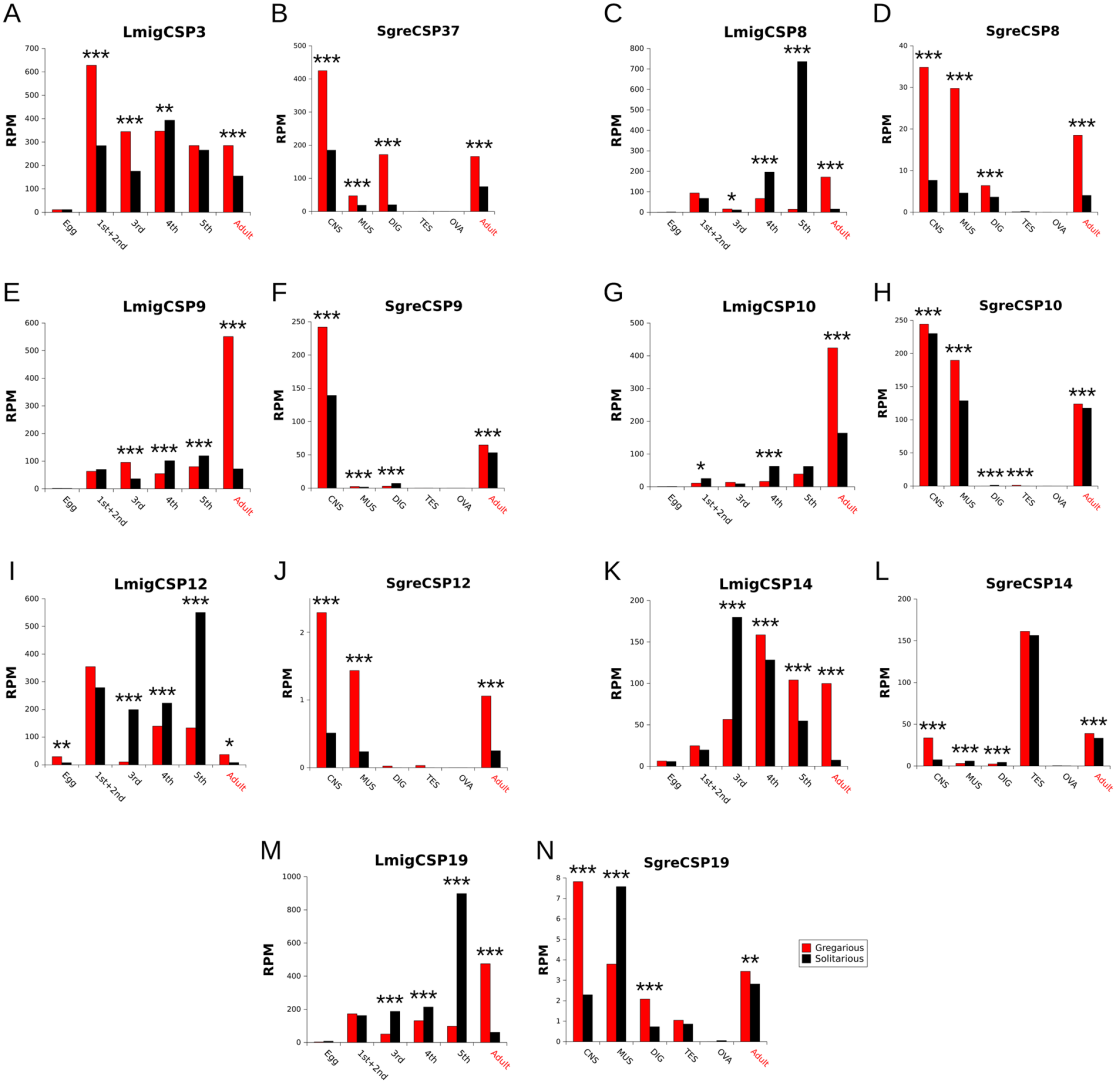
Tissue and developmental stage-specific details of the gregarious (red bars) and solitarious (black bars) expression profiles of the seven orthologous *S*. *gregaria* and *L*. *migratoria* CSP pairs that show higher expression in gregarious adult locusts in general (red text in the X axes). The Y axes represent the NGS reads mapped to the CSP per million of total mapped reads (RPM). Note that the scales of the Y axes are adapted to the expression level of each CSP. The data for orthologous pairs are in consecutive figures ((**A**–**D**) etc.)—note that *LmigCSP3* is homologous to *SgreCSP37*. The X axes in (**A**,**C**,**E**,**G**,**I**,**K**,**M**) show the analyzed developmental stages of *L*. *migratoria*, including eggs, adults with no digestive tube (see Methods) and nymphal instars (1st + 2nd = first and second instars, 3rd = third instar, 4th = fourth instar, 5^th^ = fifth instar). The X axes in (**B**,**D**,**F**,**H**,**J**,**L**,**N**) show the tissues analyzed for *S*. *gregaria* (CNS = central nervous system, MUS = muscle, DIG = digestive tube, OVA = ovaries, TES = testicles)—the data for adults in this case were the merge of the data from all the tissues but the digestive tube. The asterisks indicate the significance level of the test on normalized fold change (see Methods) after FDR correction (*0.05–0.01; **0.01–0.001 and ***0.001–0).

CSPs are involved in a plethora of biological processes some of which general to different species (detection of food, chemicals…) while others are species-specific (detection of mates, competitors…). In fact, *S*. *gregaria* and *L*. *migratoria* can mutually trigger the gregarious state in each other, although with much lower efficiency compared to the conspecific *stimuli* ^64^, and we previously reported differences in the characteristics of the phase change between both species^65^. The phase-related CSPs can therefore be species-specific, with species-dependent differential expression, or linked to the phase change in all locusts in a similar way. The latter group would be ancestral to all swarming locusts and might be of interest to the fight against locust outbreaks in general, while the species-specific CSPs and those that show species-dependent differential expression could be explored for species-specific targeting. We found that *L*. *migratoria*’s and *S*. *gregaria*’s CSPs do not share a generally conserved pattern of differential expression between phases. Yet, 14 orthologs show the same direction of over-expression in adults of both species. Interestingly, all these orthologs show over-expression in the gregarious phase (Fig. 3). One of them, *LmigCSP3*, has already been shown to be involved in *L*. *migratoria*’s phase change^36^. Its ortholog, *SgreCSP37* (90% nucleotide and 92% amino acid similarity), could therefore also be associated with the phase-change in *S*. *gregaria*—*LmigCSP3* and *SgreCSP37* might hence be involved in the phase change in locusts in general, where they may be interacting with the same molecule(s) and allowing detection of *stimuli* from non-conspecifics. The other 6 orthologous pairs that show a conserved pattern of over-expression in gregarious adults (*LmigCSP8* and *SgreCSP8*, *LmigCSP9* and *SgreCSP9*, *LmigCSP10* and *SgreCSP10*, *LmigCSP12* and *SgreCSP12*, *LmigCSP14* and *SgreCSP14* and *LmigCSP19* and *SgreCSP19*) provide more candidate genes for investigating CSP involvement in triggering and/or maintaining the gregarious phase in locusts in general.

**Figure 4:**
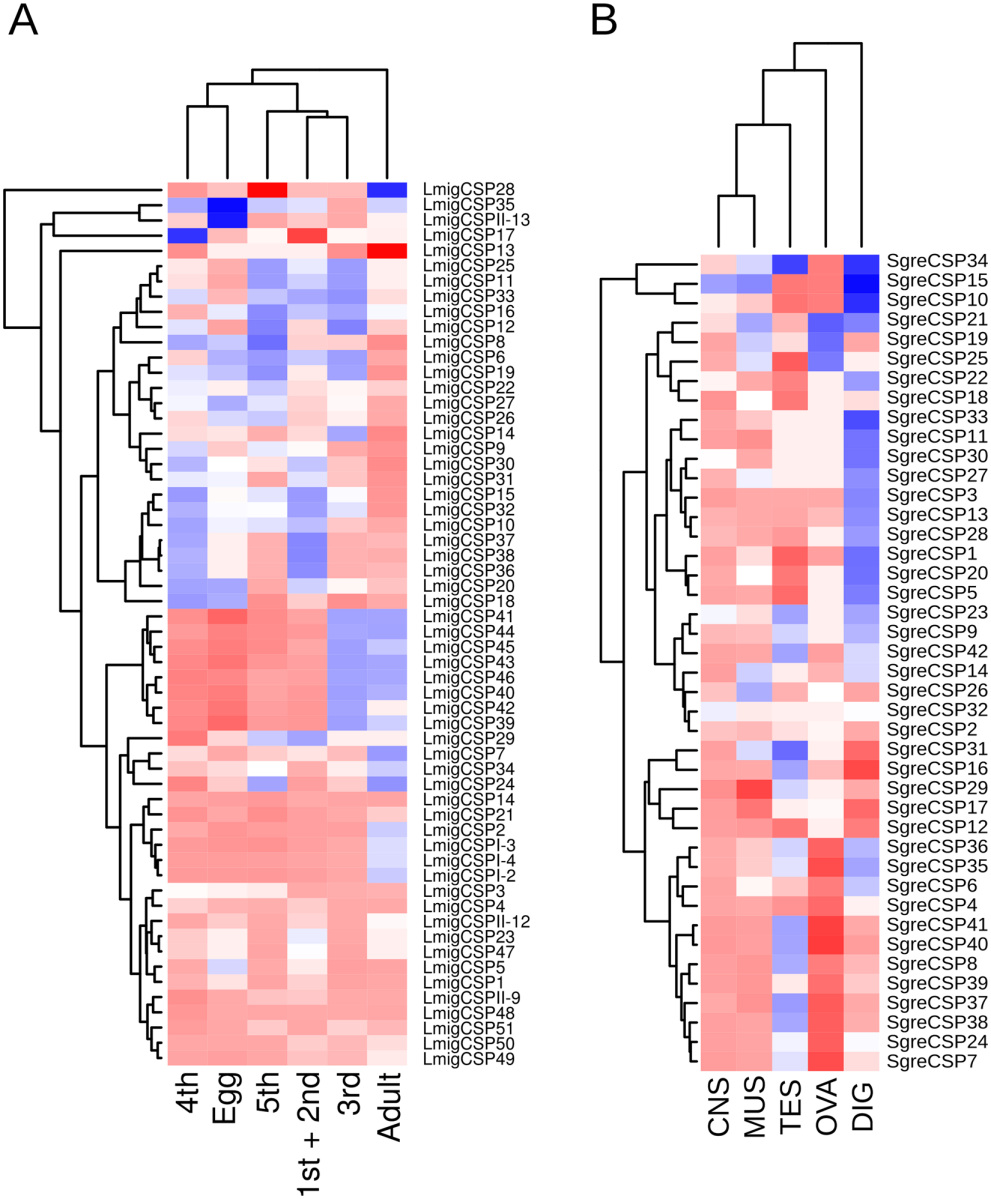
Solitarious to gregarious expression profiles of the locusts’ CSPs identified in the present work. (**A**) Comparison between *L*. *migratoria*’s nymphal instars and adults. (**B**) Comparison between *S*. *gregaria*’s tissues. The expression levels are shown as color hues proportional to the 2-based logarithm of the fold change (see Methods). Blue hues represent solitarious over-expression whereas red hues represent gregarious over-expression. The hues become lighter as the differences of the expression levels between solitarious and gregarious phases become weaker, and white hues represent non-differential expression. Dendrograms group the samples and CSPs based on similarity of the expression profiles. CNS = central nervous system, MUS = muscle, DIG = digestive tube, OVA = ovaries, TES = testicles. 1st + 2nd, 3rd, 4th and 5th in (**B**) indicate the nymphal instars.

13 clades of orthologous CSPs show opposite over-expression patterns in both species. Furthermore, 15 *S*. *gregaria*’s and 33 *L*. *migratoria*’s CSPs seem species-specific. Most of these CSPs whose presence or expression patterns are species-specific show over-expression in the gregarious phase and therefore offer a set of candidate genes of potential interest to species-specific actions on locusts (see ref. 66). Interestingly, the most modern expansion of locusts’ CSPs shows a tendency towards higher expression in the gregarious phase, no matter the instar or tissue (Fig. 4A,B). That expansion seems therefore posterior to the evolution of the phase change in locusts.

qPCR validation of the RNAseq data supports the findings on five out of six CSPs tested in *L*. *migratoria*’s adults (Figs 4A and S8A)—only *LmigCSP4* showed contradicting results. Similarly, the qPCR results on four out of six CSPs tested in *L*. *migratoria*’s 4^th^ instar nymphs supported the RNA-seq data (Figs 4A and S8C). In addition, the biased expression pattern of *SgreCSP18* towards the gregarious phase in the central nervous system was confirmed by qPCRs using *S*. *gregaria*’s adults and 4^th^ instar nymphs (Figs 4B and S8B,D). Overall, 10 out of 14 replicated qPCR testings (nine in *L*. *migratoria* and one in *S*. *gregaria*) showed the same direction of differential gene expression as the RNAseq data, four of which significant. Of the three qPCR testings that did not support the RNAseq data only one showed significant differences between gregarious and solitarious locusts (Figs 4, S8 and S9). Our RNAseq data are therefore trustable and the interpretations that we drew based on them are worth the effort of functional testing as a necessary and definitive way of validation.

In conclusion, we identified the nearly complete set of CSPs in the two main pest locust species. The fact that these organisms have a large and diversified set of CSPs is mainly due to gene duplications and speaks to the potential essential nature of these molecules for the locusts’ biology—locusts’ phase change included. Accordingly, most of these CSPs show significant differential expression between phases and, in accordance with the greater need for detection of *stimuli* in crowded conditions, most of the differentially expressed CSPs show higher expression in the gregarious phase. CSPs therefore offer potentially useful molecules for dealing with locust outbreaks. Indeed, some CSPs share similar sequences and expression patterns between species and, hence, might be of general use against all locusts, whereas others have species-specific sequences or expression patterns and might be of use for species-specific targeting. Our findings thus allow discussing the possibilities and a certain degree of speculation on the potential involvement of the CSPs in locusts’ phase change. However, our work does not allow going beyond discussing the possibilities regarding the nature of the association between some CSPs and that phase change. Our interpretations need further functional testing in order to differentiate between the differentially expressed CSPs that might be involved in triggering the gregarious state, those that might be involved in maintaining it, and those that are rather affected by it.

## Methods

### Locust rearing

*S*. *gregaria* was reared in an insectarium at the Faculty of Sciences of the University of Granada as described in ref. 65. *L*. *migratoria* specimens were reared at the Institute of Zoology of Chinese Academy of Sciences facilities as described in ref. 27.

### Sequence retrieval, characterization, filtering and assignment

Locust CSP sequences were identified following three approaches: (*i*) based on published works on locust CSPs^41, 47–49^, (*ii*) by scanning the confirmed genomic sequences from *L*. *migratoria* and *S*. *gregaria*, and (*iii*) by BLAST searches^67^ of the Sanger sequenced ESTs from *L*. *migratoria* ^27^ (EST accession numbers: CO819675 to CO832059 and CO832067 to CO865130) and *S*. *gregaria* ^24^, as well as of five *de novo* transcriptome assemblies of Illumina-sequenced RNAs from *S*. *gregaria*’s central nervous system, muscles, digestive tube, ovaries and testicles (Martín-Blázquez & Bakkali, in preparation).

We retrieved the nucleotides and amino acids sequences of the reported *L*. *migratoria*’s^27, 47–49^ and *S*. *gregaria*’s^41^ CSPs. We began by BLASTx exploration of the available scaffolds of the *L*. *migratoria*’s genome assembly version 2.4.1^29^ as queries (accession number AVCP000000000) and a local BLAST database of all the arthropod CSP protein sequences available in the NCBI database. The translated protein sequences of the genes that had positive BLASTx hits (10^−10^ E-value cut-off) were used for further confirmation by detection of the conserved cysteines pattern (C-X_6_-C-X_18_-C-X_2_-C or C-X_8_-C-X_18_-C-X_2_-C). Sequences that didn’t contain one of these patterns were discarded as non-CSPs no matter their BLAST result.

tBLASTn searches using the selected *L*. *migratoria* genomic sequences as query and our local arthropod CSP protein database allowed us to further filter the results based on the presence and orientation of the two CSP exons. We verified whether both exons of each potential CSP had coherent locations (i.e., exon 1 located upstream of exon 2) and orientation. We took as reference the structure of the CSPs reported in the genomes of the honey bee *Apis mellifera* ^56^ and silkworm *Bombyx mori* ^51^. This way we confirmed a first set of putative CSPs in the available *L*. *migratoria*’s draft genome. We also retained orphan exons 1 and 2 (exons 1 or 2 in loci where there is no exon 2 or 1, respectively) in order to check whether they might be part of partially sequenced genes. tBLASTn also allowed us to determine the exonic coordinates of the putative CSP genes in each *L*. *migratoria*’s genomic scaffold.

BLASTx searches of our local CSP protein database using *L*. *migratoria*’s ESTs from refs 27, 47–49 as queries allowed us to detect CSP transcripts. The ESTs that gave positive BLAST results (10^−10^ E-value cut-off) were further analysed using TransDecoder^68^ in order to check their amino acids’ sequences for the presence of the conserved four cysteines pattern. Redundancies at 100% identity threshold were removed using CD-HIT^69^. We assigned ESTs to genomic loci by reciprocal BLASTn searches. The assignation of an EST to a genomic locus was straightforward if the locus gives a best significant BLAST hit against an EST that does not appear as best hit against any other locus. These cases allowed us to establish a minimum BLAST identity threshold that an EST and a locus had to reach in order for them to be assigned one to the other. We determined that value for the whole CSP sequence as well as for exon 1 and exon 2 separately. This way, when various loci give best BLAST hit against the same EST, we assigned the EST to the locus that gave above threshold identity with that EST, both as a full sequence and as exon 1 and exon 2 parts. An orphan exon had to reach the threshold established for that exon in order for it to be assigned to an EST. When more than one locus (complete or orphan exon) fulfil the abovementioned thresholds, they were considered as potential gene duplicates. The loci that had no BLAST hit against the ESTs or did not reach the three BLAST identity thresholds were considered as with no evidence of transcription. Reciprocally, BLASTn search of a database of genomic loci (both complete sequences and orphan exons) using the ESTs as queries allowed us to determine the ESTs that seem alleles of the same gene. These were all the ESTs that gave above thresholds identity against the same locus or against a group of loci previously identified as gene duplicates. The ESTs that gave no acceptable hit against any genomic locus were considered as transcripts of CSP genes whose genomic loci are still unsequenced. The reciprocal BLASTn searches also allowed us to identify the exon junctions in *L*. *migratoria* CSP genes.

Given the absence of a *S*. *gregaria* draft genome, we initially performed a tBLASTn search using our local arthropod CSP protein database and the assembled contigs from our partial *S*. *gregaria*’s genomic DNA library^55^ as query. As to the ESTs, we used the ones from ref. 24 as well as ten NGS (Illumina HiSeq. 2000 paired end) *de novo* assembled solitarious and gregarious transcriptomes from the CNS, digestive tube, thoracic and hind leg muscles, ovaries and testicles (over 500 million sequencing reads). The downstream analyses were as described for *L*. *migratoria*.

Before we went further with the analyses (phylogeny and expression), we established the relationships between the inferred *L*. *migratoria* and *S*. *gregaria* CSPs in order to detect and remove any remaining redundancies. We built a nucleotide phylogeny using the full set of *L*. *migratoria*’s genomic CSP loci and ESTs and identified the different clades of putative alleles based on the tree and BLAST results. We then calculated the minimum within-clade sequence identity value and used it as threshold above which nucleotide sequences of the same species could be considered as alleles of the same CSP gene. We only took into account the clades that did not contain more than one genomic sequence (no gene duplicates) for calculating the identity values, due to the uncertainties in assigning transcripts to gene duplicates. We then calculated the pairwise sequence identities for *S*. *gregaria*’s CSP ESTs and removed redundancy (transcripts of potential alleles of the same gene) based on the abovementioned threshold.

Sequence alignments were carried out using the MAFFT-LINSI option of MAFFT v7^70^, as it focuses on aligning a conserved core region and gives less importance to the non-conserved flanking regions. Maximum likelihood trees were built using PhyML v3.1^71^, with 1000 bootstrap iterations, and the PhyML Newick output format was obtained using the online version of the interactive Tree of Life tool iTOL^72^. The CD-HIT-EST command of CD-HIT^69^ was used with the lowest identity possible (80%) in order to obtain sequence identity matrices, identify the minimum identity threshold between the sequences of the *L*. *migratoria* clades, and remove all but one sequence of each set of *S*. *gregaria* ESTs that show higher identity than that threshold (putative alleles of the same gene).

We had to deal with two additional issues in the case of *S*. *gregaria*: detecting the exon junctions is not as straightforward as in the case of *L*. *migratoria*, and some of the assembled ESTs from the NGS libraries may be chimeric (*i*.*e*., assembly artifacts). We used the exonic sequences of *L*. *migratoria*’s CSPs for tracing the exon junctions on *S*. *gregaria*’s transcripts. Since this method did not work in most of the cases, we built BLAST databases using the identified exon 1 and exon 2 sequences of *S*. *gregaria*’s ESTs and carried out BLASTn searches using the ESTs whose exon junctions were not previously located. The searches were repeated after updating both exon BLAST databases, by addition of the newly identified exons, until BLASTn cessed to produce new significant results. We then built consensus sequences from the identified exon 1 and exon 2 alignments and aligned them to each EST that still had no located exon junction. This way we successfully characterised the exon junctions for all *S*. *gregaria*’s CSP transcripts.

As to the sequences that might have resulted as assembly artifact, we generated two separate trees using exon 1 and exon 2 nucleotide sequences from all the CSPs identified in *S*. *gregaria* and *L*. *migratoria*. We then extracted the identity matrices of *L*. *migratoria*’s exon 1 and exon 2 sequences and identified the highest identity value for each exon excluding the potentially duplicated loci (i.e., the high identity between recently duplicated CSP copies might impede detection of *S*. *gregaria* exons with marginally higher distances). These values were used as respective exon sequence identity thresholds for attributing exons to CSP variants. We similarly calculated the identity values between the exons of *S*. *gregaria*’s CSP ESTs that were incongruently placed in exon 1 and exon 2 trees and their nearest neighbour sequences in the corresponding trees. To assign an incongruent EST to a clade or discard it as potentially chimeric, we had to deal with three possible cases: (*i*) when both of the identity values between the exons of that EST and their nearest sequence in the tree were below-threshold (see below), (*ii*) when the identity value between one of the two exons of that EST and its nearest sequence in the tree was below or within-threshold, whereas the identity value between the other exon and its nearest sequence in the tree was above or within-threshold, and (*iii*) when the two exons of that EST showed above or within-threshold identities to their respective nearest neighbour sequences in the trees. We considered the putative *S*. *gregaria* ESTs that fit the first two cases as not chimeric and assigned them to a clade according to the phylogenetic location of their full length sequences (see below). An EST would be chimeric if it fit the third case. The alignments and phylogenies were made as described earlier.

### Locusts’ CSPs evolution

To establish the evolutionary relationships between *L*. *migratoria*’s and *S*. *gregaria*’s CSPs we built maximum likelihood phylogenies using full length sequences. The alignments and phylogenies were made as described earlier. In addition, we translated the nucleotide sequences and aligned them using the MAFFT-LINSI command of MAFFT v7^70^. We used the online version of ProtTest 2.4^73^ for obtaining the fittest amino acid substitution model, which we used for building a maximum likelihood phylogeny using PhyML v3.1^71^ with 1000 bootstrap iterations. The reason for building both nucleotides and amino acids trees was to confirm the position of the incongruent ESTs and to check whether the functional products followed a similar evolutionary path as their DNA source.

We also built an amino acids phylogeny of locust and non-locust CSPs in order to infer their overall evolution. We searched the NCBI protein database and retrieved all the CSP sequences that belong to insect species whose number of CSP copies is confirmed. We thus had CSPs from the fruit fly *Drosophila melanogaster*, the mosquito *Anopheles gambiae*, the red flour beetle *Tribolium castaneum*, the silkworm *Bombyx mori*, the honey bee *Apis mellifera*, the pea aphid *Acyrthosiphon pisum* and the head louse *Pediculus humanus*. The sequences were selected based on ref. 40, excluding those marked as pseudogenes or incomplete. The phylogenies were built as described earlier. The very nature of the multi-copies sequences and sequences from gene families makes rooting the trees with a single external sequence from a related species ineffective (i.e., a single outgroup sequence does not guarantee ancestry between all the analyzed sequences and the outgroup). One way to deal with that is to use as outgroups all the sequences of the same gene family from a related species. We used all the CSPs from the water flea *Daphnia pulex* (crustacean). This way we could also locate the locusts’ last CSP ancestry point by outgrouping at the Arthropoda phylum level.

To standardize the nomenclature of the locusts’ CSPs, we revised (without renaming) the names that were attributed to locust CSPs elsewhere and we named the CSPs reported here based on their phylogenetic proximity to known insect CSPs. We named all *L*. *migratoria*’s CSPs first then we named *S*. *gregaria*’s based on homology. To avoid introducing more noise and/or confusion, we did not change the names of the CSPs that were already named elsewhere, even when we considered it pertinent, and we retained the chronologically first nomenclature for CSPs that were reported elsewhere under different names.

We calculated the CSPs’ nucleotide diversity and non-synonymous to synonymous substitution rates (K_a_/K_s_). For that, we separately aligned the coding regions belonging to each clade, using MAFFT as described above, and we used DNAsp v.5^74^ for calculating the nucleotide diversity estimators π and θ, with their respective standard errors, and the number of synonymous and non-synonymous substitutions. The mean value of the pairwise K_a_/K_s_ ratios were calculated for each of the phylogenetic clades that contained more than two CSP sequences.

### Differential gene expression

For comparing the expression of the CSPs between gregarious and solitarious locusts, we mapped the *L*. *migratoria* and *S*. *gregaria* RNA-seq reads to their respective transcriptomes. We used the sequencing reads obtained by Chen *et al*.^26^ from gregarious and solitarious *L*. *migratoria*’s eggs (SRA accessions SRR058432 and SRR058451, respectively), 1st and 2nd nymphal instars combined (SRR058446 and SRR058452), 3rd nymphal instar (SRR058447 and SRR058453), 4th nymphal instar (SRR058492 and SRR058457), 5th nymphal instar (SRR058448 and SRR058454) and adult bodies that were devoid of their digestive tubes (SRR058455 and SRR058449), as well as the solitarious and gregarious Illumina Hiseq. 2000 Paired End reads that are currently being analyzed in our laboratory for comparative transcriptomics works on adult *S*. *gregaria* tissues (central nervous system, digestive tube, muscles, ovaries and testicles). As *L*. *migratoria* reference transcriptome, we used the published gene set derived from predicted transcripts in that species’s draft genome (http://159.226.67.243/download.htm) and the additional CSPs identified in the present work. For *S*. *gregaria*, we separately used *de novo* assemblies form our five NGS libraries and complemented each assembly with the CSPs of the other assemblies. We used BWA version 0.6.2^75^ for mapping the sequencing reads to the respective reference transcriptomes and, after cleaning the unmapped reads, we summarized the read counts using HTSeq^76^ as described in ref. 55. Read counts were normalized by the total number of mapped reads to the corresponding library and 2-based logarithm of the fold change of the normalized read counts (comparing solitarious against gregarious) was used for generating heatmaps and their corresponding dendrograms with the default command in R v2.15.0 environment^77^.

We summed all the read counts from all the *S*. *gregaria* gregarious tissues and all the read counts from all the *S*. *gregaria* solitarious tissues in order to obtain total read counts for solitarious and gregarious adult bodies that did not include their digestive tubes. The total counts were then normalized as described before. We did not include *S*. *gregaria*’s guts libraries in this overall expression analysis because *L*. *migratoria*’s sequencing data in ref. 26 did not include the digestive tube—because it is too contaminated with DNAs and RNAs from microorganisms and foods.

A CSP was notoriously expressed in the testicles (see Results). Since the sequenced tissue for that library should include the ejaculatory bulb, we checked whether that CSP is a locust homolog of the ejaculatory bulb specific protein III (EBP3, accession number U08281)—a protein that seems to be homologous to CSPs^41, 44, 48^. We built an amino acids phylogeny adding EBP3 sequences from the pea aphid (*ApisEBP3*, accession number NP_001156287.1), the red floor beetle (*TcasEBP3*, accession number XP_008196341.1) and the fruit fly (*DmelEBP3*, accession number NP_524966.1). We used the same methodology and software as detailed earlier and we included an odorant binding protein from *L*. *migratoria* (*LmigOBP*, accession number ACI30696.1) as outgroup.

qPCRs were carried out for complementing and double-checking the RNA-seq results. We designed primers from the non-conserved regions of the CSPs in order to avoid non-specific amplification. This way we obtained primers for six putative *L*. *migratoria* CSPs (Table S13). We used RNAzol RT (Molecular Research Center, Inc.) for extracting total RNAs from the heads of eight *L*. *migratoria* adults (four gregarious and four solitarious), eight *L*. *migratoria* 4th instar nymphs (four gregarious and four solitarious) and eight *S*. *gregaria* 4^th^ instar nymphs (four gregarious and four solitarious) as well as from the central nervous system tissues of *S*. *gregaria* adults (five gregarious and five solitarious). Residual gDNA was removed by DNAse I (Sigma-Aldritch) treatment and 16 cDNA libraries were separately synthesized in 20 µL reaction volumes using 1 µg of each total RNA and Superscript III RT kit (Invitrogen). qPCRs were carried out using 5 µL of the SensiMix SYBR kit mix (Bioline), 5 µL of 1:50 dilutions of the synthesized cDNA, 1 µL of each primer (10 pmol) and 3 µL of RNAse-free distilled water. The cycling conditions were 95 °C for 10 min, then 40 cycles of 94 °C for 15 s, 60 °C for 15 s, and 72 °C for 15 s. The plate-reads were taken after each extension (i.e., 72 °C) step. Melting curves were built between each 72 °C and 95 °C step, with a plate-read every 1 °C, in order to verify that only a single DNA product was amplified. We used DNA Engine Peltier Thermal Cycler with a Chromo4 continuous fluorescence detector (Bio Rad) and the quantification was carried out using the delta Ct method, according to ref. 78. We used the GeNorm software (Primer Design, Ltd., Southampton University, Highfield Campus, Southampton Haunts, UK) and the housekeeping genes in refs 79 and 80 for selecting the most stable ones between locust phases. These were tubulin A1 for *S*. *gregaria* and ribosomal protein 49 for *L*. *migratoria* (primers in Supplementary Table S13). Each experimental and housekeeping gene was tested in triplicate for each cDNA.

## Electronic supplementary material

Suplementary Figures

Suplementary Tables

Ka/Ks matrix
