# Supplementary material for: Evolution, expression and association of the chemosensory protein genes with the outbreak phase of the two main pest locusts
Source: Sci Rep. 2017 Jul 27;7:6653. doi: 10.1038/s41598-017-07068-0 (PMC5532218; doi:10.1038/s41598-017-07068-0)
Supplement: Supplementary file 1 — Suplementary Figures [file 41598_2017_7068_MOESM1_ESM.pdf]

Evolution, expression and association of the chemosensory protein genes with the outbreak phase of the two main pest locusts

Martín-Blázquez, R., Chen, B., Kang, L. and Bakkali, M.*
mbakkali@ugr.es

Supplementary figures

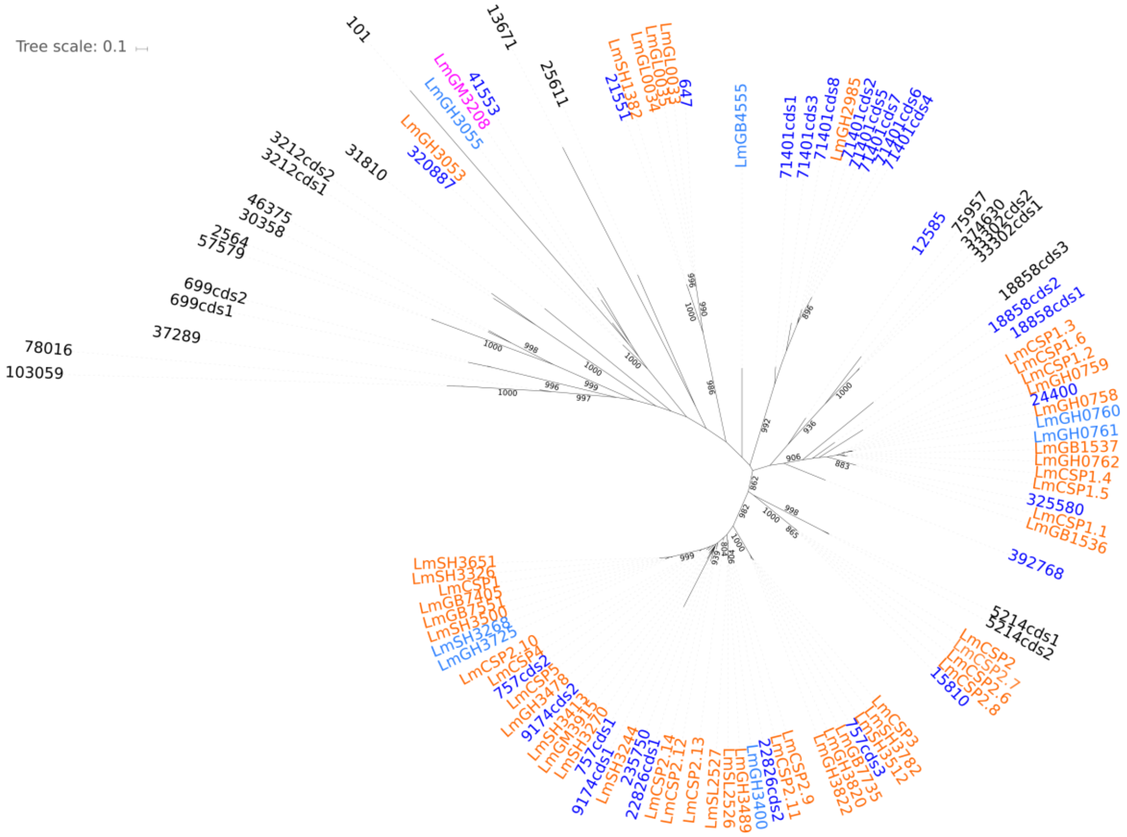

Figure S1. Maximum likelihood phylogenetic tree of the nucleotide sequences of the *L. migratoria* CSP genes and ESTs. The CSPs whose sequences were identified in *L. migratoria* genome are in blue (with positive BLAST result against ESTs) or black (without positive BLAST result against ESTs), *L. migratoria* ESTs are in orange, loci derived from ESTs are in light blue and the only CSP EST for which no genomic locus has been identified is in pink. Only branch supports that are higher than 75 % are shown in their respective branches. The green bubbles mark the sequence clusters used for establishing the identity threshold for removal of redundant alleles from *S. gregaria* CSP transcripts.

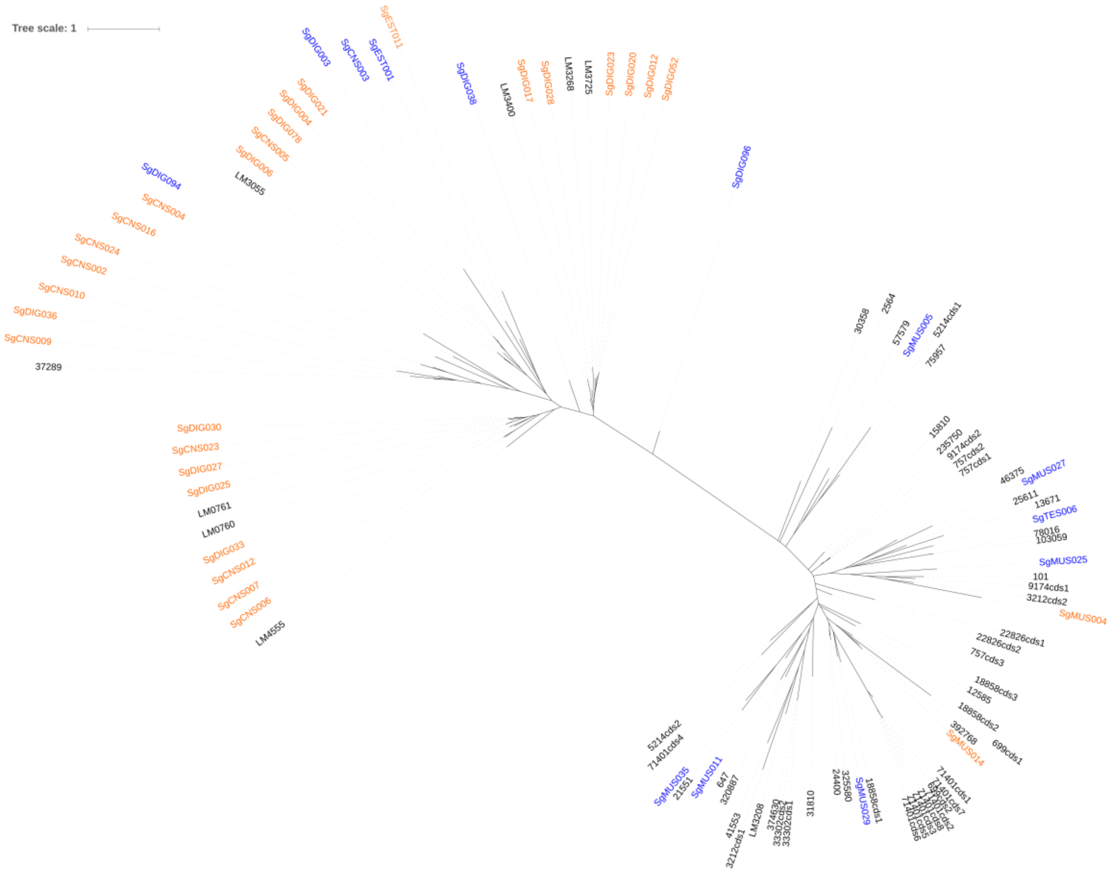
Tree scale: 1
SgEST011
SgDIG003
SgCNS003
SgEST001
SgDIG021
SgDIG004
SgDIG078
SgCNS005
SgDIG006
LM3055
SgDIG038
LM3400
SgDIG017
SgDIG028
LM3268
LM3725
SgDIG023
SgDIG020
SgDIG012
SgDIG052
SgDIG096
SgDIG094
SgCNS004
SgCNS016
SgCNS024
SgCNS002
SgCNS010
SgDIG036
SgCNS009
37289
SgDIG030
SgCNS023
SgDIG027
SgDIG025
LM0761
LM0760
SgDIG033
SgCNS012
SgCNS007
SgCNS006
LM4555
30358
2564
57579
SgMUS005
5214cds1
75957
15810
235750
9174cds2
757cds2
757cds1
46375
SgMUS027
25611
13671
SgTES006
78016
103059
SgMUS025
101
9174cds1
3212cds2
SgMUS004
22826cds1
22826cds2
757cds3
18858cds3
12585
18858cds2
392768
SgMUS014
699cds1
5214cds2
71401cds4
SgMUS035
21551
SgMUS011
647
320867
41553
3212cds1
LM3208
31810
24400
325580
SgMUS029
18858cds1
71401cds1

A

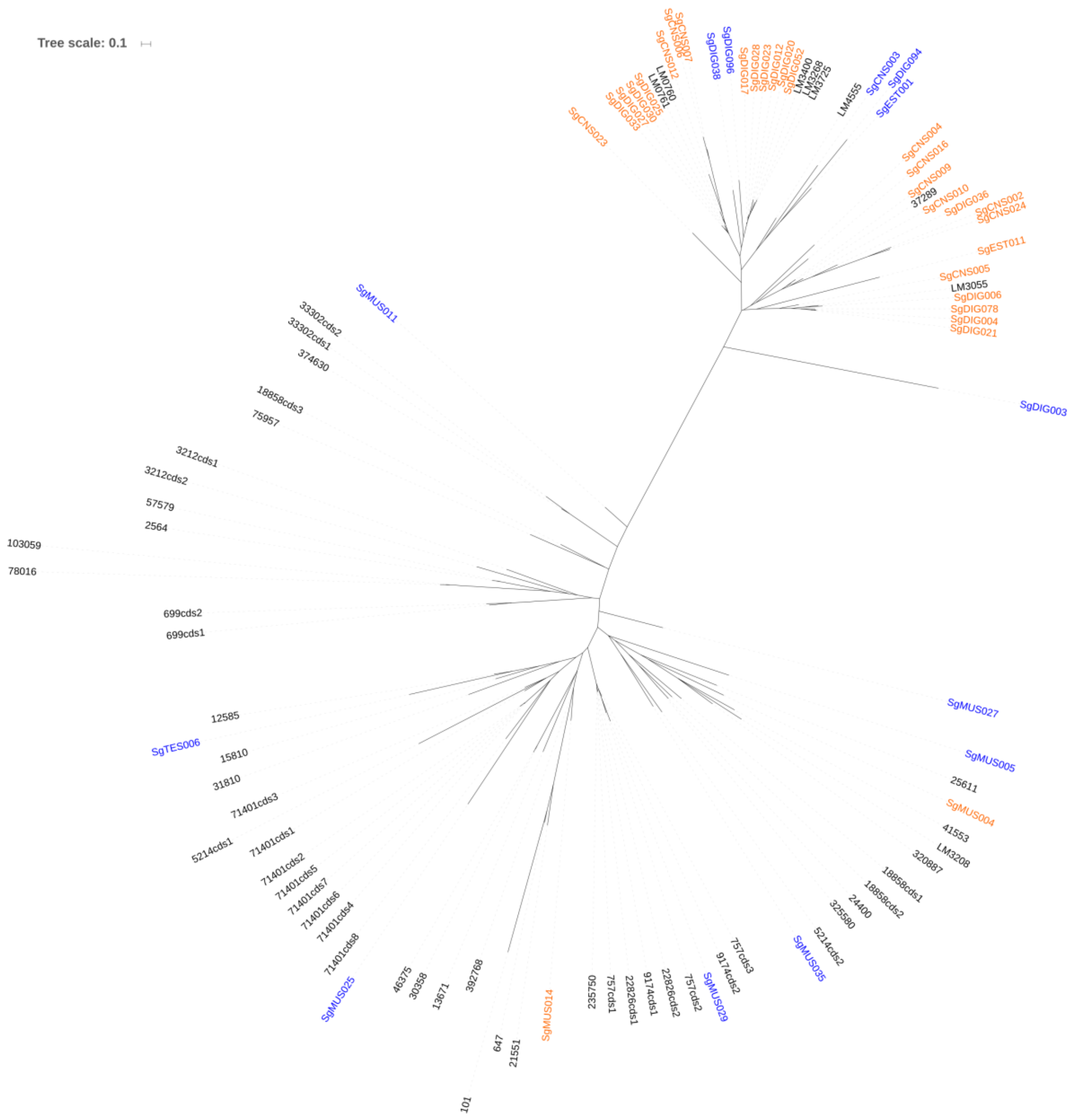
Tree scale: 0.1
SgCNS007
SgCNS008
SgCNS012
LM0760
LM0761
SgDIG025
SgDIG030
SgDIG027
SgDIG033
SgCNS023
SgDIG038
SgDIG096
SgDIG017
SgDIG028
SgDIG023
SgDIG012
SgDIG020
SgDIG052
LM3400
LM3268
LM3725
LM4555
SgCNS003
SgDIG094
SgEST001
SgCNS004
SgCNS016
SgCNS009
37289
SgCNS010
SgDIG036
SgCNS002
SgCNS024
SgEST011
SgCNS005
LM3055
SgDIG006
SgDIG078
SgDIG004
SgDIG021
SgDIG003
SgMUS027
SgMUS005
25611
SgMUS004
41553
LM3208
320887
18858cds1
18858cds2
24400
325580
5214cds2
SgMUS035
757cds3
9174cds2
SgMUS029
757cds2
22826cds2
9174cds1
22826cds1
757cds1
235750
SgMUS014
21551
647
101
392768
13671
30358
46375
SgMUS025
71401cds8
71401cds4
71401cds6
71401cds7
71401cds5
71401cds2
71401cds1
5214cds1
71401cds3
31810
15810
SgTES006
12585
699cds1
699cds2
78016
103059
2564
57579
3212cds2
3212cds1
75957
18858cds3
374630
33302cds1
33302cds2
SgMUS011

B

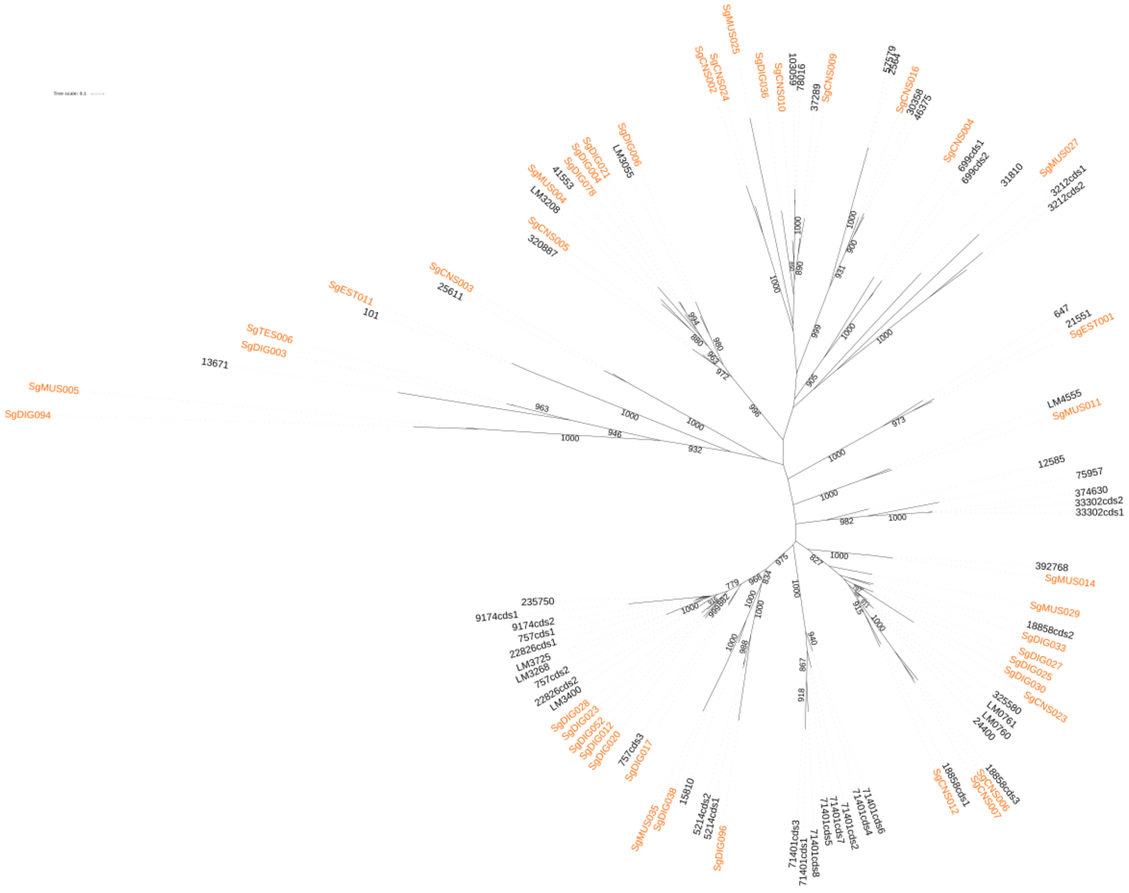
SgMUS025
SgCNS024
SgCNS002
SgDIG036
SgCNS010
78016
37289
SgCNS009
57579
SgCNS016
30358
46375
SgCNS004
699cds1
699cds2
31810
SgMUS027
3212cds1
3212cds2
SgDIG006
LM3055
SgDIG021
SgDIG004
SgDIG078
41553
SgMUS004
LM3208
SgCNS005
320887
SgCNS003
25611
SgEST011
101
SgTES006
SgDIG003
13671
SgMUS005
SgDIG094
647
21551
SgEST001
LM4555
SgMUS011
12585
75957
374630
33302cds2
33302cds1
392768
SgMUS014
SgMUS029
18858cds2
SgDIG033
SgDIG027
SgDIG025
SgDIG030
SgCNS023
325580
LM0761
LM0760
24400
18858cds3
SgCNS006
SgCNS007
18858cds1
SgCNS012
71401cds6
71401cds4
71401cds2
71401cds7
71401cds5
71401cds3
71401cds1
71401cds8
5214cds2
5214cds1
SgDIG096
15810
SgDIG038
SgMUS035
757cds3
SgDIG017
235750
9174cds1
9174cds2
757cds1
22826cds1
LM3725
LM3268
757cds2
22826cds2
LM3400
SgDIG028
SgDIG023
SgDIG052
SgDIG012
SgDIG020

C

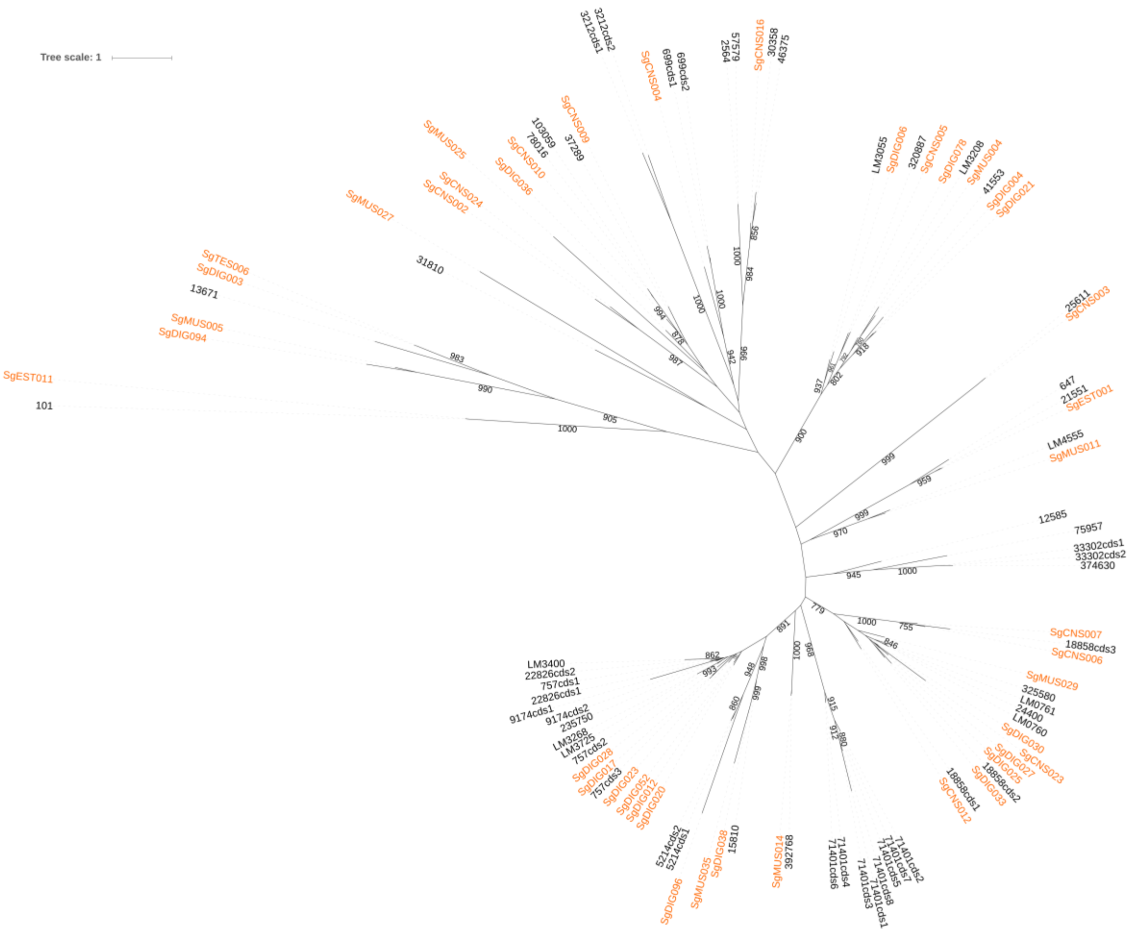

D

Figure S2. Maximum likelihood phylogenetic tree of the nucleotide (A-C) and amino acid (D) sequences of exon 1 (A), exon 2 (B) and whole (C) *L. migratoria* and *S. gregaria* CSPs. *L. migratoria* CSPs are in black and *S. gregaria* transcripts in orange. Incongruently placed *S. gregaria* exon sequences are in blue. The branch length scale is at the top of the trees. Only branch support values that are higher than 75 % are shown in their respective branches.

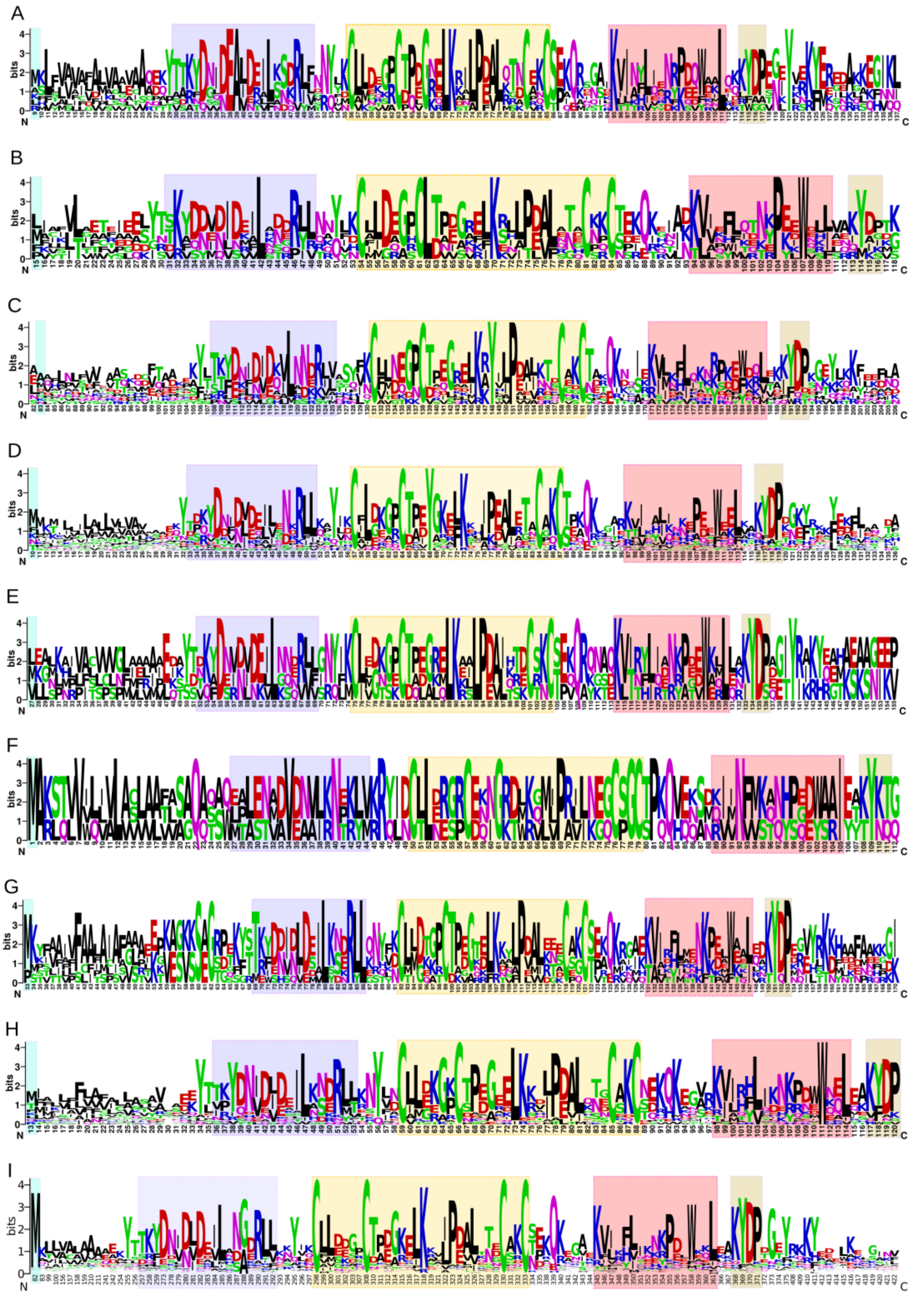

Figure S3. Sequence logo reflecting amino acid conservation along the positions of the alignment from *Anopheles gambiae* (A), *Apis mellifera* (B), *Acyrthosiphon pisum* (C), *Bombyx mori* (D), *Drosophila melanogaster* (E), *Daphnia pulex* (F), *Pediculus humanus* (G), *Tribolium castaneum* (H), and all these species plus *L. migratoria* and *S. gregaria* (I). The five conserved sections of the alignment (consensus initial methionine, signal peptide conserved region, cysteine box, leucine-isoleucine-valine-methionine conserved region and KYDP region) are boxed.

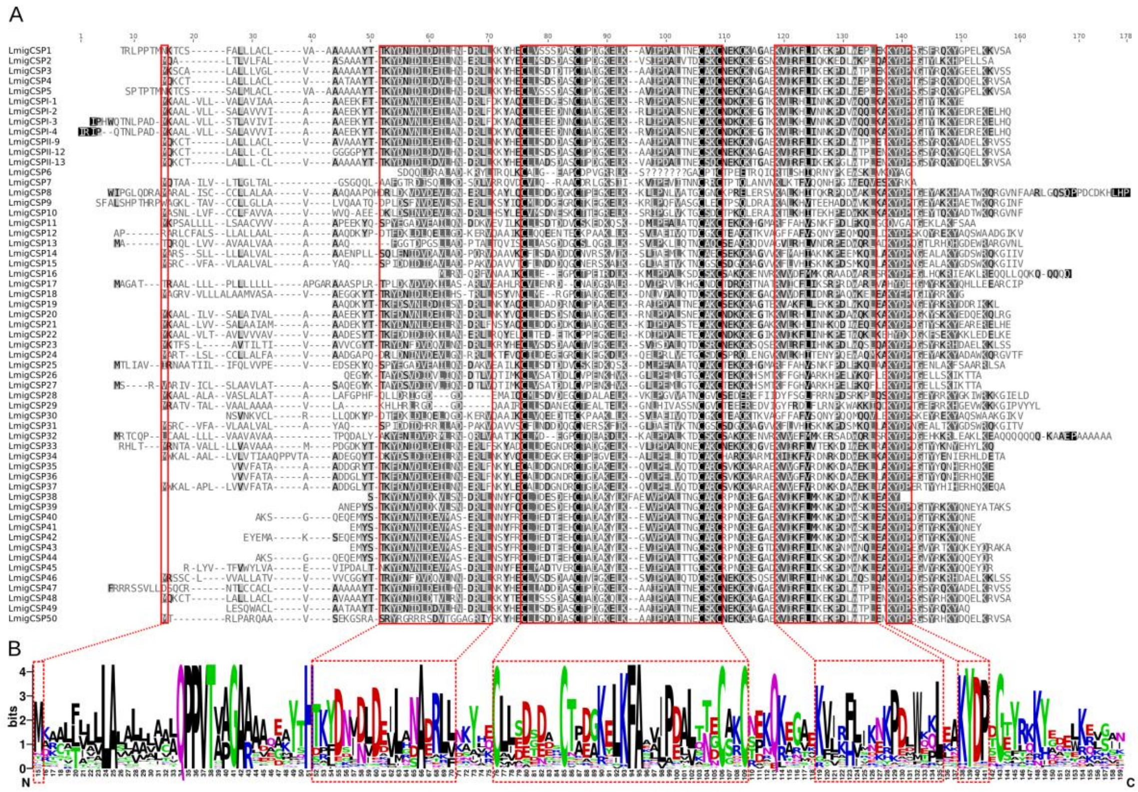

Figure S4. Alignment (A) and sequence logo (B) of *L. migratoria* CSP amino acid sequences. Similarity is proportionally represented in the alignment in greyscale hues, being white a non-conserved position and black a fully conserved position. The height of the logo at each position reflects conservation of that position. Five conserved sections (consensus initial methionine, signal peptide conserved region, cysteine box, leucine-isoleucine-valine-methionine conserved region and KYDP region) are boxed in both figures.

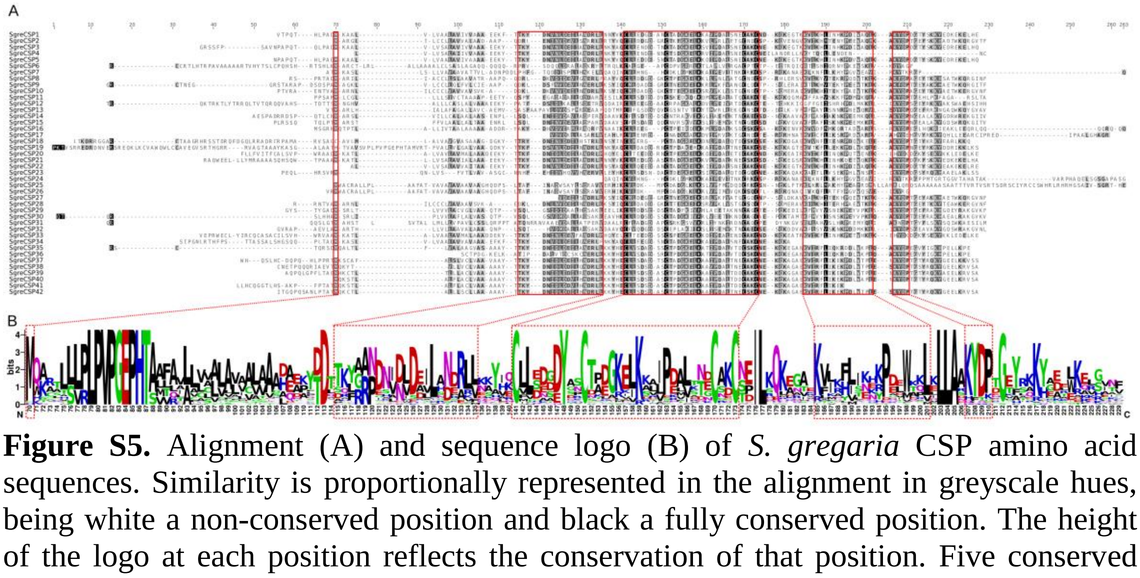

Figure S5. Alignment (A) and sequence logo (B) of *S. gregaria* CSP amino acid sequences. Similarity is proportionally represented in the alignment in greyscale hues, being white a non-conserved position and black a fully conserved position. The height of the logo at each position reflects the conservation of that position. Five conserved sections (consensus initial methionine, signal peptide conserved region, cysteine box, leucine-isoleucine-valine-methionine conserved region and KYDP region) are boxed in both figures.

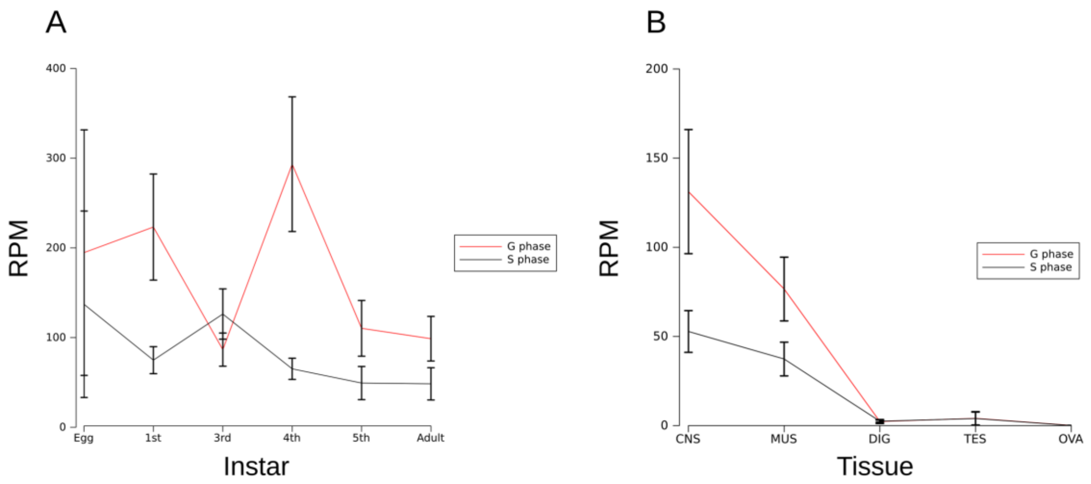

Figure S6. Distribution of the mean reads per million of total mapped reads (RPM) from all the CSPs identified in *L. migratoria* (A) and *S. gregaria* (B).

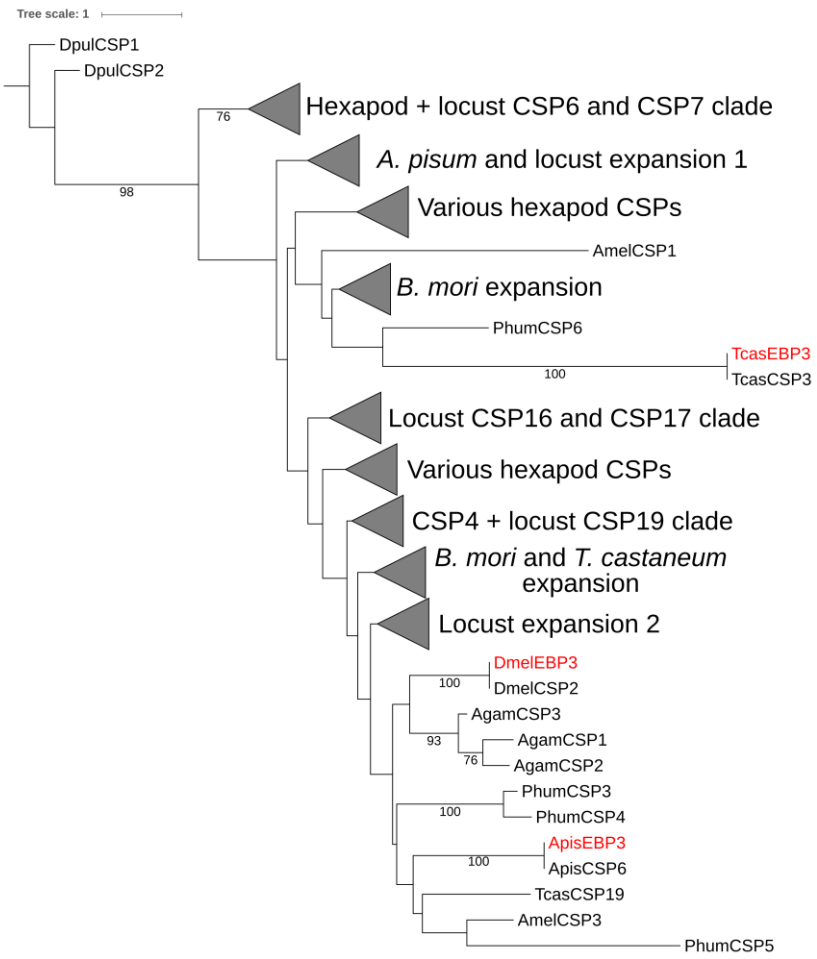

Figure S7. Maximum likelihood phylogenetic tree of the amino acid sequences of locust CSPs and insect EBP3 with *L. migratoria* OBP sequence as outgroup. Non-relevant branches were collapsed for clarity.

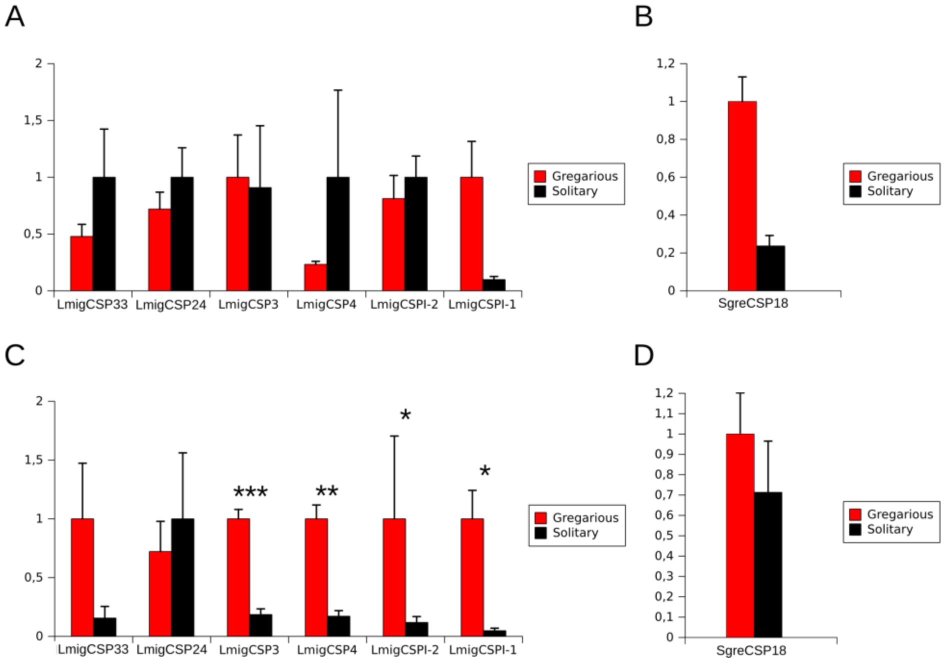

Figure S8. Standardized qPCR results of the expression of six *L. migratoria* and one *S. gregaria* CSP in adults (A and B) and nymphs (C and D). The standardization consisted in dividing by the highest value of the expression levels, so the maximum value for each CSP sequence is 1. Red bars represent gregarious expression levels and black bars represent solitarious expression values. *t*-tests were performed to obtain signification and, in case of significantly difference, asterisks are placed over the bars.

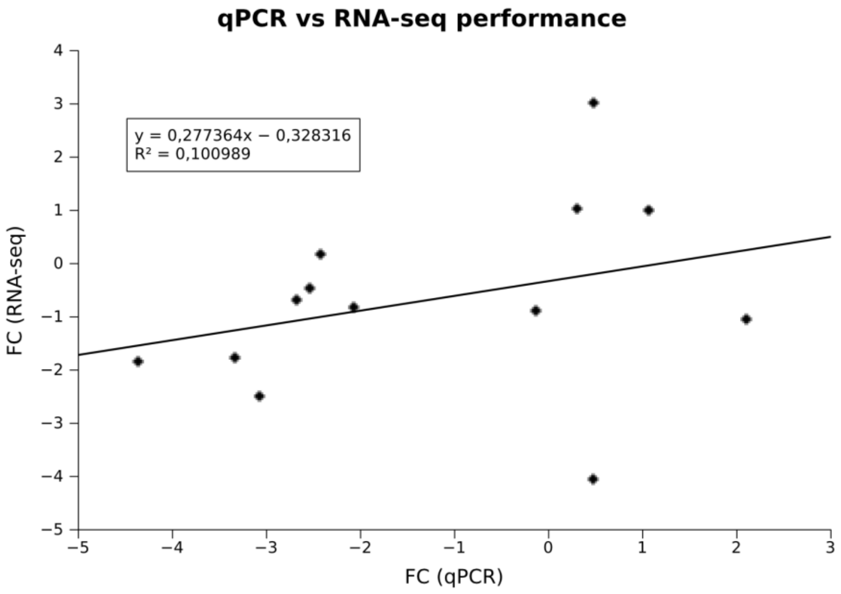

Figure S9. Comparison between the RNA-seq and qPCR results on CSPs' differential expression between solitarious and gregarious *L. migratoria*. The fold change was calculated as detailed in Material and Methods. Pearson regression coefficients were $R^2 = 0.203$ for adults and $R^2 = 0.268$ for 4th instar nymph.
